# *Lycium ruthenicum* as a Functional Food Resource: Chemical Composition, Nutritional Value, Health Benefits, and Biological Activities

**DOI:** 10.3390/nu18193160

**Published:** 2026-09-25

**Authors:** Yujin Liu, Ziqi Meng, Sihan Liu, Chenyi Fan, Yin Cheng, Yujun Liu, Xicheng Zhang, Chengzhu Cao, Ying Ma, Ying Zhang, Youwei Du, Huiya Ma

**Affiliations:** 1 Department of Basic Medical Science, Qinghai University Medical College, Xining 810001, China; 2 College of Plant Protection, Northwest A&F University, Yangling 712100, China

**Keywords:** *Lycium ruthenicum*, anthocyanins, functional food, nutraceuticals, biological activities, metabolic regulation

## Abstract

*Lycium ruthenicum* Murr., commonly known as black goji berry, is a solanaceous shrub distributed in saline-alkaline desert environments of the Qinghai–Tibet Plateau and other arid regions. Its growing interest as a functional food resource is closely related to a rich nutritional profile and a phytochemical pattern that differs from that of the common red goji berry (*Lycium barbarum*). The most distinctive feature is the abundance of anthocyanins, particularly petunidin-derived compounds, which contribute to its dark fruit color and many of its reported biological properties. This review brings together evidence on the chemical composition, nutritional value, biological activities, and food-processing applications of *L. ruthenicum*. Anthocyanins, polysaccharides, fatty acids, amino acids, minerals, and trace elements are discussed in relation to antioxidant, anti-aging, neuroprotective, metabolic-regulatory, and immunomodulatory effects reported in current studies. Most of these health-relevant effects are currently supported by chemical/biochemical assays, in vitro studies, and animal models, whereas direct human evidence remains limited. Processing strategies, including drying, extraction, fermentation, encapsulation, and intelligent packaging, are considered alongside emerging genomic evidence related to stress tolerance and quality formation. The available evidence indicates that *L. ruthenicum* is a promising functional food and nutraceutical resource, although its bioavailability, safety, mechanisms of action, and clinical relevance still require more rigorous evaluation.

## 1. Introduction

*Lycium ruthenicum* Murr., commonly known as black goji berry or black wolfberry, is a long-lived thorny shrub belonging to the family Solanaceae. It is mainly distributed in saline-alkaline, arid, and high-ultraviolet environments, including the Qinghai–Tibet Plateau and the desert regions of Xinjiang and Gansu, China [1,2,3]. Owing to its strong tolerance to drought, salinity, and other environmental stresses, *L. ruthenicum* has developed distinctive adaptive characteristics that are closely associated with its phytochemical accumulation and nutritional properties [3,4]. Traditionally, it has been used in Tibetan and Uygur medicine and has gradually gained attention as both a medicinal food and a modern functional food resource [1]. This dual identity reflects the concept of medicine-food homology, which has long been embedded in traditional Chinese dietary and therapeutic culture.

Among the bioactive constituents of *L. ruthenicum*, anthocyanins are considered the most characteristic compounds. These natural pigments are responsible for the dark purple-black color of the fruit and contribute substantially to its antioxidant potential [1,5,6].

Compared with the commonly consumed red goji berry (*Lycium barbarum*), *L. ruthenicum* exhibits a distinct phytochemical profile, particularly characterized by abundant anthocyanins, including petunidin-derived compounds [7]. In contrast, *L. barbarum* is mainly recognized for its carotenoid components, especially zeaxanthin dipalmitate, which contribute to its characteristic orange-red coloration [7]. Although both species belong to the genus *Lycium* and share traditional medicinal and dietary applications, their dominant phytochemical constituents differ, suggesting that they represent complementary functional food resources rather than interchangeable products. Previous studies have reported that these anthocyanins exhibit strong free-radical-scavenging capacity and may contribute to a range of biological activities, such as antioxidant, anti-aging, neuroprotective, metabolic-regulatory, and immunomodulatory effects [6]. Therefore, *L. ruthenicum* is increasingly regarded as a promising natural source of functional ingredients for foods, beverages, nutraceuticals, and health-related products [8,9].

The practical value of *L. ruthenicum* is constrained by several issues that recur across the literature. Wild resources are geographically restricted, while standardized large-scale cultivation is still developing. Fruit quality also changes markedly with geographical origin, environmental exposure, cultivation practices, and postharvest handling, which makes raw-material consistency difficult to guarantee. Processing introduces another layer of uncertainty because anthocyanins are sensitive to light, heat, oxygen, and pH, leading to color fading, nutrient loss, and reduced product stability [10,11,12,13]. These linked problems show why resource evaluation, compositional analysis, biological assessment, and processing research need to be considered together rather than as separate topics.

Against this background, the present review summarizes recent advances in *L. ruthenicum* as a functional food resource. The discussion moves from botanical and resource characteristics to chemical composition, nutritional value, biological activities, processing technologies, genomic resources, and future applications. Particular attention is given to quality control, anthocyanin stability, product development, and the question of how experimental evidence can be translated into reliable food and health-related products.

Across the reviewed studies, one methodological issue is especially noticeable: chemical profiling, activity testing, and processing evaluation are often carried out independently. Bringing these strands together is necessary because a compound that is abundant in fresh fruit may still lose value if it is unstable during drying, poorly absorbed after digestion, or difficult to standardize in a commercial product [8].

As summarized in Figure 1, the value of *L. ruthenicum* can be understood through five interconnected dimensions: phytochemical composition, particularly petunidin-derived anthocyanins, polysaccharides, and seed-oil bioactives [5,14,15]; reported biological activities and product-development potential [3,16]; processing strategies, including encapsulation and intelligent packaging technologies [17,18]; and omics-based insights into WRKY/MYB regulation, anthocyanin biosynthesis, and stress adaptation [19,20,21,22].

## 2. Review Methodology and Evidence Appraisal

This review was conducted as a narrative review with a systematic literature search and transparent evidence appraisal. The search and selection process followed the PRISMA 2020 guidelines.

### 2.1. Literature Search

Two databases were searched: PubMed and Web of Science Core Collection. The search was performed on 26 July 2026, with a time span from 26 July 2016 to 26 July 2026. Only English-language original articles and reviews were considered. The search combined species terms (“*Lycium ruthenicum*”, “black goji”, “black wolfberry”) with terms related to composition, bioactivity, processing, and genomics, including anthocyanin, polysaccharide, polyphenol, flavonoid, composition, processing, bioactivity, antioxidant, anti-inflammatory, neuroprotective, metabolic, immune, gut microbiota, genomic, transcriptomic, and “functional food”.

The full search strings for each database are provided in Supplementary Materials Note S1. Records were exported, deduplicated, and screened. A total of 539 records were identified, and 516 remained after deduplication.

### 2.2. Inclusion and Exclusion Criteria

Eligible studies included original research focusing on *Lycium ruthenicum* as the primary experimental material, providing valid data regarding its chemical composition, nutritional characteristics, processing technologies, product development, genomic and transcriptomic profiles, or biological functions. In vitro cell experiments, in vivo animal studies, and human-related investigations were all acceptable. In addition, high-quality systematic and narrative reviews closely associated with the bioactive mechanisms, processing optimization, and functional food applications of *Lycium ruthenicum* were also included to support comprehensive thematic analysis.

Studies were excluded if they were irrelevant to *Lycium ruthenicum* or failed to provide definite material sources and sample information. Publications with repeated datasets, incomplete methodological descriptions, or insufficient experimental data were excluded. Moreover, studies focusing neither on nutritional composition, biological activity, nor functional food development were eliminated. Conference abstracts, editorials, letters, errata, and other non-original or non-research articles were also excluded to guarantee the reliability and integrity of the reviewed evidence.

### 2.3. Screening and Data Extraction

Two researchers independently screened titles and abstracts, followed by full-text assessment. Disagreements were resolved by a third researcher. Data extracted from each included study included first author, year, journal, country, study type, fruit origin, cultivar, processing method, chemical components, analytical methods, biological activities, mechanisms, main outcomes, and reported limitations.

### 2.4. Quality Assessment and Evidence Appraisal

Study quality was assessed according to study design. Chemical characterization studies were evaluated based on sample authentication, standard substance application, method validation, reproducibility, and compound structural identification. Cell and animal experiments were assessed for controls, randomization, blinding, sample size, dose–response relationships, and mechanistic evidence. Human studies were evaluated with respect to study design, randomization, blinding, placebo control, and validity of endpoints. Reviews were assessed for search transparency and internal quality appraisal.

Given the heterogeneous nature of the available evidence on *L. ruthenicum*, including chemical assays, in vitro models, animal studies, and limited human investigations, evidence was categorized into four tiers according to the proximity of the experimental system to human physiological relevance. Tier 1 represented human evidence, including clinical and intervention studies; Tier 2 represented in vivo evidence obtained from animal models; Tier 3 represented in vitro biological evidence derived from cell-based or tissue models; and Tier 4 represented chemical or biochemical evidence, such as antioxidant capacity assays and enzyme-inhibition studies. Importantly, these evidence tiers indicate the translational proximity of evidence rather than methodological quality, and lower-tier evidence should not be interpreted as inherently inferior. Instead, the framework was used to facilitate critical interpretation of biological activities and to avoid overgeneralization of preclinical findings to human health effects. Table 1 presents the evidence classification framework adopted in this review.

Given the substantial heterogeneity of included studies in terms of experimental design, raw material sources, processing conditions and outcome indicators, results were synthesized narratively rather than combined via meta-analysis. Limitations of this review include incomplete database coverage, language restrictions, publication bias, inter-study heterogeneity, and citation lag for recently published literature.

## 3. Nutritional and Phytochemical Composition

The nutritional value and potential health benefits of *Lycium ruthenicum* Murr. arise from a complex phytochemical profile rather than from a single dominant constituent. Unlike *Lycium barbarum*, which is commonly associated with carotenoids and polysaccharides, *L. ruthenicum* is marked by abundant polyphenols, especially anthocyanins, together with polysaccharides, fatty acids, alkaloids, amino acids, minerals, and trace elements [14,23]. These compounds shape the fruit’s color, flavor, nutritional quality, processing behavior, and reported biological activities. The following section therefore examines the main nutritional and phytochemical groups with attention to structural features, quantitative variation, extraction, stability, and environmental regulation.

### 3.1. Anthocyanins: Signature Pigments of L. ruthenicum

Anthocyanins are generally regarded as the most characteristic bioactive compounds in *L. ruthenicum* and represent an important chemotaxonomic feature that distinguishes this species from other *Lycium* [1]. These water-soluble flavonoid pigments are responsible for the typical dark purple-black color of the fruit and are closely associated with its antioxidant capacity and free-radical-scavenging activity [3,7]. Biosynthetically, anthocyanins are derived from the phenylpropanoid and flavonoid pathways and are mainly stored in vacuoles. Compared with the relatively simple anthocyanin profiles reported in many edible berries, the anthocyanins of *L. ruthenicum* are characterized by extensive glycosylation and acylation. These structural modifications may improve pigment stability and influence their physicochemical properties and biological functions [5,24].

The composition summarized in Table 2 illustrates the chemical complexity behind the dark fruit color of *L. ruthenicum*. Petunidin derivatives dominate the anthocyanin profile, whereas delphinidin and malvidin derivatives occur at lower levels. Identification has mainly relied on chromatographic and mass spectrometric techniques, including HPLC-DAD-ESI-MS, UPLC-Triple-TOF/MS, and MS-based structural analysis [25,26,27], which is important because many of these compounds differ only in acylation pattern or isomeric configuration.

#### 3.1.1. Structural Diversity and Identification

Chromatographic and spectrometric analyses have revealed a structurally diverse anthocyanin profile in *L. ruthenicum*. Petunidin derivatives constitute the dominant group of anthocyanins, followed by delphinidin and malvidin derivatives in relatively lower proportions [25,26,27]. This petunidin-rich profile is one of the major chemical features distinguishing *L. ruthenicum* from other anthocyanin-rich berries, such as blueberries and blackcurrants, in which malvidin- or cyanidin-based anthocyanins are often more abundant [5,29].

The major anthocyanins reported in *L. ruthenicum* are highly glycosylated and frequently acylated with phenolic acids, particularly *p*-coumaric acid. A representative compound is petunidin-3-*O*-[rhamnopyranosyl-(trans-*p*-coumaroyl)-glucopyranoside]-5-*O*-glucopyranoside, which has been repeatedly identified as a key anthocyanin in the fruit [12,24]. Other reported derivatives include petunidin-3-*O*-rutinoside-5-*O*-glucoside, petunidin-3-*O*-*p*-coumaroylrutinoside-5-*O*-glucoside, delphinidin-based derivatives, and minor malvidin derivatives [25,26,27,29].

Recent advances in chromatographic separation, including mixed-mode reversed-phase/strong anion-exchange stationary phases, have enabled more precise discrimination of cis- and trans-isomers of acylated anthocyanins [26]. The presence of acyl groups is particularly important because acylated anthocyanins generally exhibit greater resistance to light, heat, and pH variation than non-acylated anthocyanins. This structural feature may partly explain the relatively high color stability of *L. ruthenicum* anthocyanin extracts in food-related systems [30].

#### 3.1.2. Quantification and Extraction Yields

Reported total anthocyanin values are best interpreted in the context of the analytical method used. The total anthocyanin content of *L. ruthenicum* fruits is high, but published values vary with cultivar or population, geographical origin, maturity stage, sample preparation, extraction solvent, and expression unit [31,32]. Some studies report values of approximately 600–1800 mg/100 g fresh weight; comparisons across studies, however, require caution because results may be expressed as fresh weight, dry weight, extract weight, or cyanidin-3-glucoside equivalents [31,32].

Extraction efficiency is another key factor affecting the recovery and stability of anthocyanins from *L. ruthenicum*. Conventional extraction methods frequently rely on acidified ethanol or methanol systems, whereas emerging green extraction strategies have been developed to reduce organic solvent use and improve anthocyanin preservation. For example, beta-cyclodextrin-assisted aqueous extraction has been reported to enhance anthocyanin yield and stability by forming inclusion complexes with anthocyanin molecules, thereby reducing degradation during processing [33,34]. Compared with *L. barbarum*, which contains little or no anthocyanins, *L. ruthenicum* represents a much richer source of these pigments and may be comparable to or higher than some commercial anthocyanin-rich berries, depending on the materials and analytical conditions used [35].

#### 3.1.3. Analytical Limitations and Standardization of Anthocyanin Characterization

Most anthocyanin assignments for *L. ruthenicum* rely on HPLC-DAD-ESI-MS and UPLC-Triple-TOF-MS data, yet such mass-spectrometry-based identification remains tentative rather than definitive structural confirmation. These analytical workflows cannot unambiguously assign acylation positions, exact glycosidic linkage sites, or distinguish cis- and trans-isomers for acylated anthocyanins, even with mixed-mode chromatographic approaches that offer only partial isomer resolution. The shortage of commercially available authentic reference standards further weakens confidence in structural assignments.

Quantitative datasets are similarly constrained by methodological inconsistencies. Varied normalization bases (fresh, dry, or extract weight) and the widespread adoption of cyanidin-3-glucoside equivalents introduce systematic bias, which is particularly relevant given the petunidin-dominant anthocyanin profile of *L. ruthenicum*. Extraction variables including solvent composition, acidification, temperature, and β-cyclodextrin addition and other additives further alter anthocyanin recovery, hindering reliable cross-study comparisons. Collectively, these constraints highlight the need for unified extraction and quantification protocols supported by structurally validated reference markers to support robust quality assessment and industrial utilization of these pigments.

#### 3.1.4. Environmental Regulation of Anthocyanin Biosynthesis

Metabolomic studies show that anthocyanin-related metabolites, including malvidin-3-*O*-rutinoside-5-*O*-glucoside and other flavonoid derivatives, can increase under salinity stress [21,36]. Comparative transcriptomic analyses between *L. ruthenicum* and more salt-sensitive *Lycium* species reveal that salt exposure leads to differential accumulation of flavonoids and anthocyanins between the two species [37].

### 3.2. Phenolic Acids and Non-Anthocyanin Flavonoids

Although anthocyanins are the most characteristic polyphenols in *L. ruthenicum*, non-anthocyanin phenolic compounds also contribute substantially to its nutritional quality and biological activity. These compounds mainly include phenolic acids and flavonols, which together form an important part of the total phenolic content of the fruit [38]. Beyond direct antioxidant capacity, they may interact with anthocyanins and other phytochemicals, thereby influencing pigment stability, flavor characteristics, and health-promoting potential.

#### Profile of Phenolic Acids

The content and composition of phenolic acids in *L. ruthenicum* are influenced by geographical origin, environmental conditions, maturity stage, and postharvest processing. Comparative studies have suggested that samples from different production areas, such as Qinghai, Xinjiang, and Gansu, may exhibit distinct phenolic acid profiles [39]. For example, berries from the Qaidam Basin in Qinghai have been reported to contain relatively higher levels of chlorogenic acid and caffeic acid than those from some other regions, which may be related to local microclimatic conditions, soil characteristics, and stress-related secondary metabolism. However, further standardized comparative studies are still required to clarify the relationship between ecological factors and phenolic acid accumulation [39].The major non-anthocyanin phenolic acids identified in *L. ruthenicum* fruits are summarized in Table 3.

**Table 3:** Major non-anthocyanin phenolic acids identified in *Lycium ruthenicum* fruits.

Phenolic Acid	Structural Formula	Key Characteristics	References
Chlorogenic acid	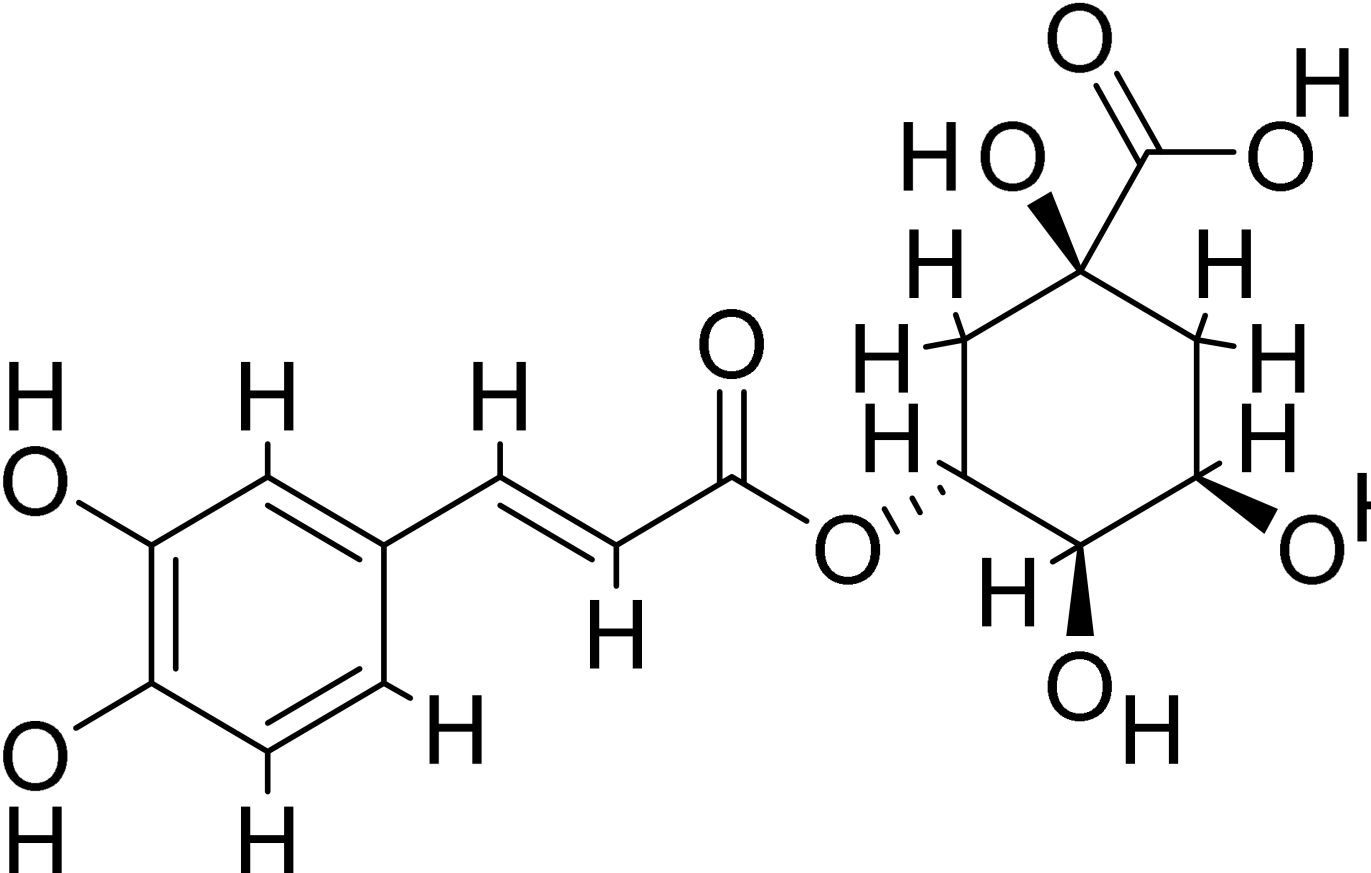	One of the most abundant non-anthocyanin phenolic acids reported in *L. ruthenicum*; contributes to total phenolic content and antioxidant potential.	[40]
Caffeic acid	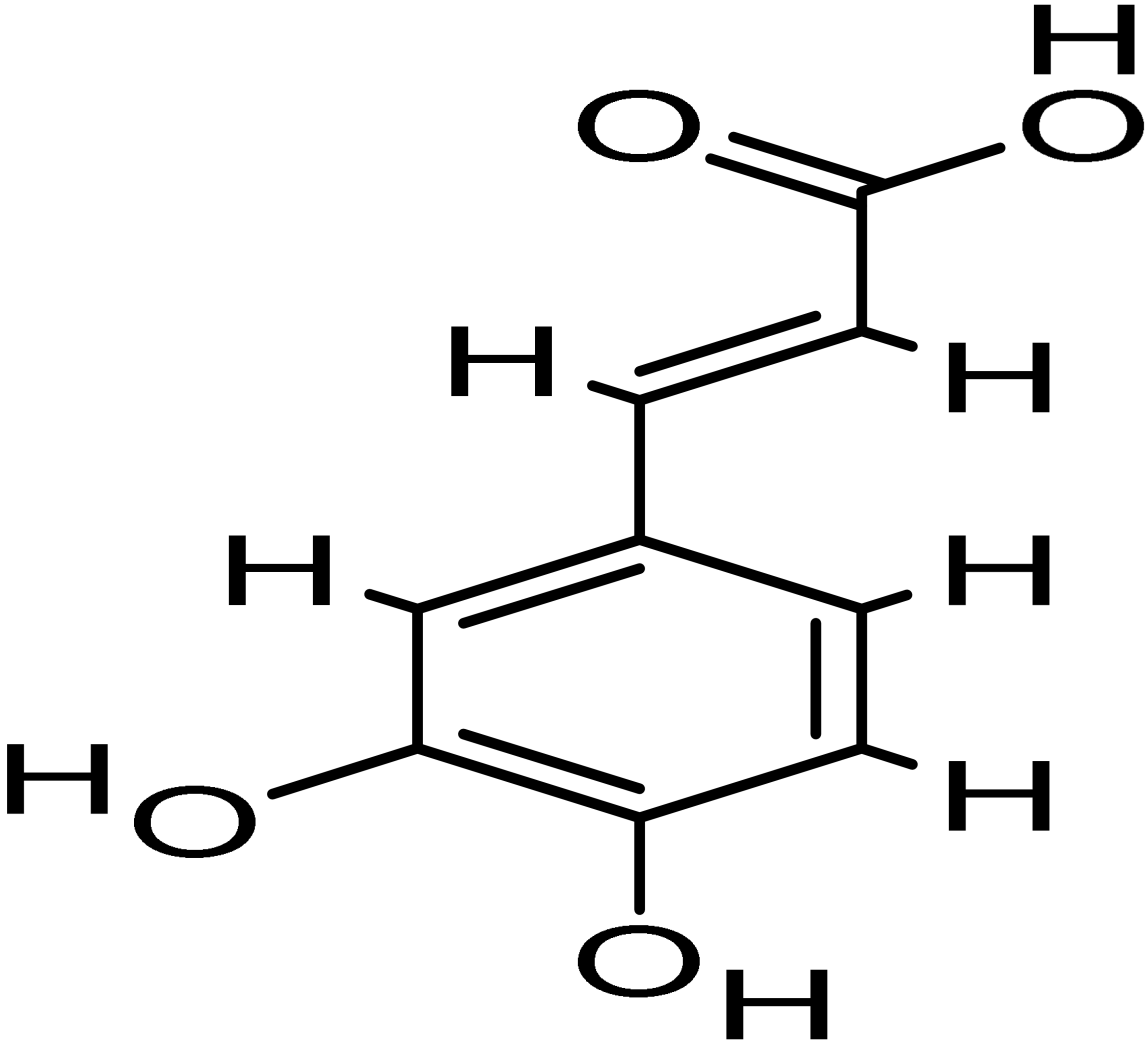	A hydroxycinnamic acid associated with lignin and flavonoid biosynthetic pathways.	[40]
*p*-Coumaric acid	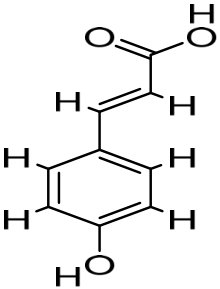	Structurally linked to anthocyanin acylation and may contribute to anthocyanin stability.	[40]
Gallic acid	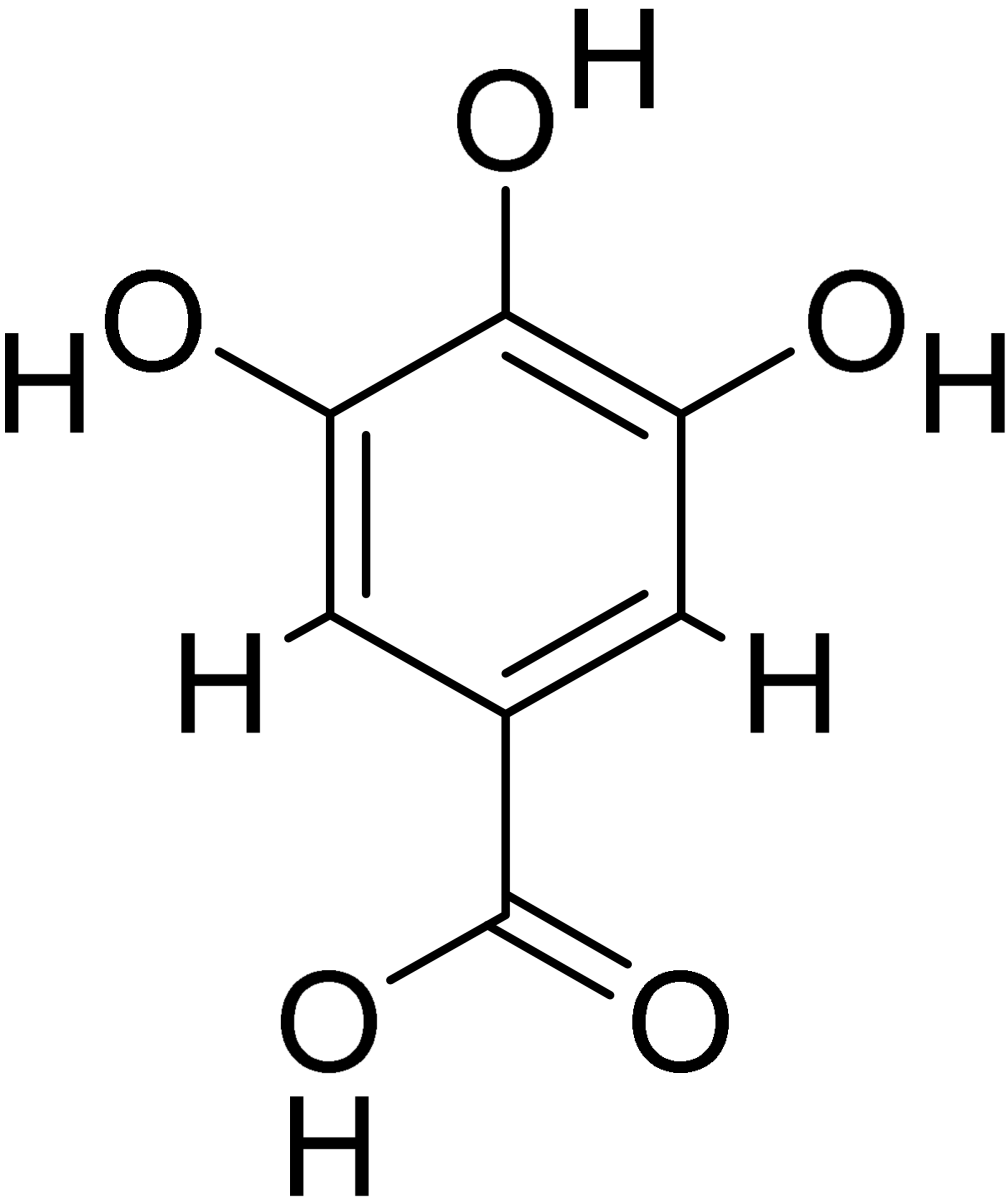	Detected at relatively low levels but may contribute to antioxidant properties.	[40]
Ferulic acid	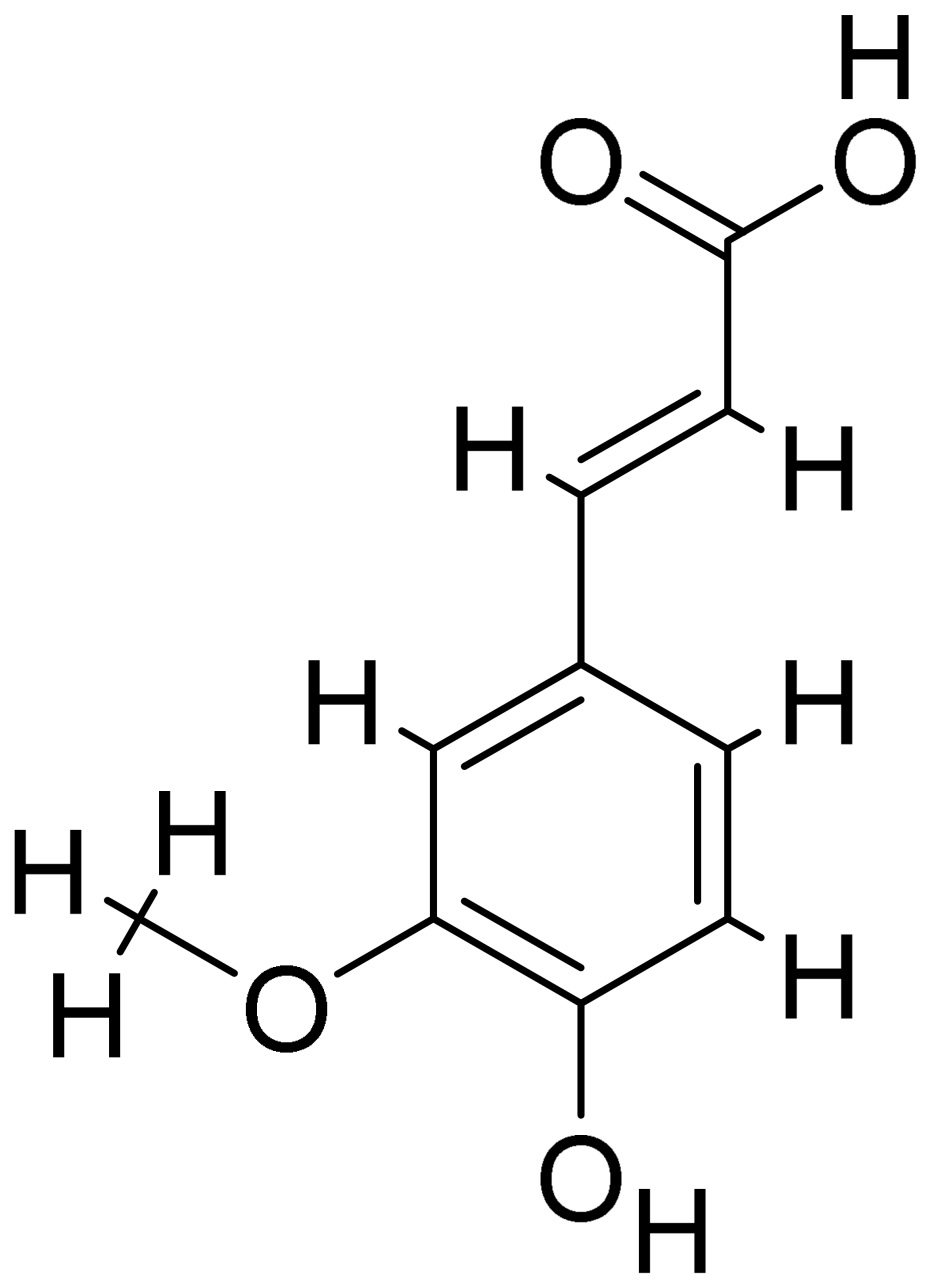	A phenolic acid present in smaller quantities and associated with antioxidant and protective effects.	[41]

Recent phytochemical investigations have further expanded the known polyphenolic profile of *L. ruthenicum*. Several newly identified glycosidic polyphenols have been isolated from the fruit and reported to exhibit bioactivities related to neuroprotection [42]. Some of these compounds showed monoamine oxidase B inhibitory activity and improved cell viability in oxidative stress-related cellular models [42]. These findings suggest that non-anthocyanin flavonoids and glycosides may also contribute to the biological activities of *L. ruthenicum*, rather than functioning merely as minor accompanying constituents. Nevertheless, current evidence is still mainly derived from chemical characterization and in vitro experiments; therefore, further studies are needed to confirm their bioavailability, metabolism, dose–response relationships, and in vivo relevance.

In addition to phenolic acids, *L. ruthenicum* contains a variety of flavonols, mainly in glycosylated forms. Representative flavonols include quercetin, kaempferol, and myricetin derivatives, such as rutin and nicotiflorin [16]. These compounds may act together with anthocyanins and phenolic acids to enhance the antioxidant capacity of the fruit and may also contribute to its anti-inflammatory, metabolic-regulatory, and neuroprotective activities [43].

### 3.3. Polysaccharides: Structural Complexity and Bioactivity

*Lycium ruthenicum* polysaccharides (LRPs) are important water-soluble macromolecules and represent one of the major nutritional and functional constituents of the fruit. Previous studies have reported that polysaccharides account for approximately 3.5–6.5% of the dried fruit weight, although the exact content may vary depending on geographical origin, extraction conditions, maturity stage, and analytical methods [14,44]. Compared with polysaccharides from *Lycium barbarum* (LBPs), LRPs show distinct structural characteristics, including differences in monosaccharide composition, molecular weight distribution, glycosidic linkages, branching patterns, and uronic acid content [44,45,46]. These structural differences are closely associated with their physicochemical properties and may contribute to their specific biological activities. Therefore, a clearer understanding of LRP heterogeneity is necessary before their structure-function relationships and potential applications can be properly evaluated.

#### 3.3.1. Monosaccharide Composition and Molecular Weight Distribution

As summarized in Table 4, LRPs should not be regarded as a single uniform material, but rather as a group of structurally diverse polysaccharide fractions. Representative fractions isolated from *L. ruthenicum* fruits differ in extraction method, purification procedure, monosaccharide composition, and molecular weight, all of which may affect solubility, conformation, and bioactivity.

The molar ratios of monosaccharides differ markedly among purified LRP fractions, such as LRP1-S2, LRP4-A, LRGP1, LRGP3, LRGP5, LRP-S2A, and LRP3-S1 (Table 4). In many reported fractions, galactose and arabinose are relatively abundant, suggesting the possible presence of arabinogalactan-type structural domains. Such structural features may be relevant to the immunomodulatory and antioxidant activities reported for LRPs [46,47]. However, because extraction, purification, hydrolysis, and detection methods vary among studies, direct comparison of different polysaccharide fractions should be made cautiously.

To provide a clearer overview of the structural diversity of LRPs, Table 4 summarizes representative polysaccharide fractions isolated from *L. ruthenicum* fruits, including their extraction methods, purification procedures, monosaccharide composition, molecular weight, and references.

**Table 4:** Representative polysaccharide fractions isolated from *Lycium ruthenicum* fruits and their structural characteristics.

Designation of Polysaccharide	Extraction Method	Extraction Conditions	Separation and Purification Methods	Structural Formula	Monosaccharide Composition (Molar %)	Molecular Weight (kD)	References
LRP1-S2	Hot water extraction	Water-to-raw material ratio: 6.67 mL/g, extraction temperature: 100 °C, extraction time: 4 h	DEAE Sepharose Fast Flow and Sephacryl S100 HR columns	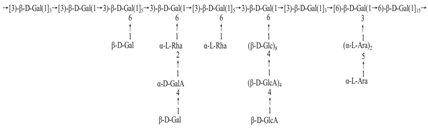	Gal 46, Ara 40, Rha 5, Glc 4, GlcA 2, Man 2, GalA 1	17	He et al., 2021 [48]
LRP4-A	Hot water extraction	Extraction temperature: 80 °C Extraction time: 2 h	DEAE-cellulose anion exchange and Sephadex G100 columns	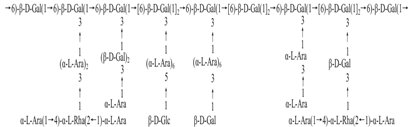	Gal 48, Ara 43, Rha 6, Glc 3	105	Lv et al., 2013 [49]
LRGP1	Hot water extraction	Water-to-raw material ratio: 4 mL/g, extraction temperature: 70 °C, extraction time: 2 h	DEAE-cellulose anion-exchange and Sephadex G100 columns	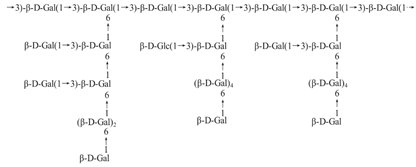	Gal 44, Ara 45, Rha 3, Glc 4, Man 3, Xyl 1	56.2	Peng, Lv, Xu, et al., 2012 [50]
LRGP3	Hot water extraction	Water- to-raw material ratio: 4 mL/g, extraction temperature: 70 °C, extraction time: 2 h	DEAE-cellulose anion-exchange and Sephadex G100 columns	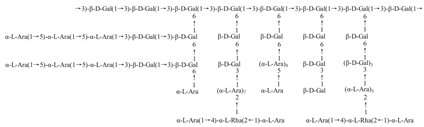	Gal 39, Ara 57, Rha 4	75.6	Peng, Song, Lv, et al., 2012 [51]
LRGP5	Hot water extraction	Water-to-raw material ratio: 4 mL/g, extraction temperature: 70 °C, extraction time: 2 h	DEAE-cellulose anion-exchange and Sephadex G-100 columns	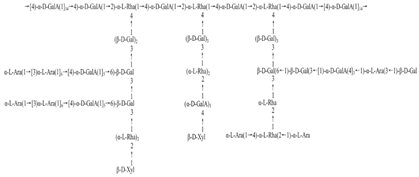	Gal 13, Ara 24, Rha 10, GalA 49, Xyl 5	137	Peng, Xu, et al., 2014 [52]
LRP-S2A	Hot water extraction	Extraction temperature: 80 °C, extraction time: 2 h	DEAE Sepharose Fast Flow and Sephacryl S500 HR columns	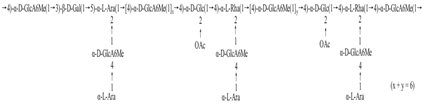	Gal 5, Ara 20, Rha 9, Glc 25, GlcA 41,	2650	Wang, Liu, et al., 2020 [44]
LRP3-S1	Hot water extraction	Water-to-raw material ratio:6.67 mL/g, extraction temperature: 100 °C, extraction time: 4 h	DEAE Sepharose Fast Flow and Sephacryl S300 HR columns	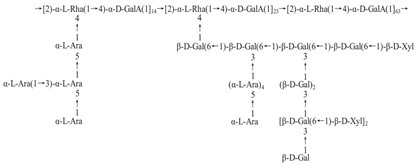	Gal 27, Ara 25, Rha 14, GalA 18, Xyl 16	114.8	Zhang et al., 2019 [53]

#### 3.3.2. Structural Features and Structural Modifications

LRPs exhibit considerable structural heterogeneity, which is reflected in their monosaccharide composition, molecular weight distribution, glycosidic linkage patterns, branching degree, and uronic acid content [46]. This heterogeneity is closely related to differences in extraction procedures, purification strategies, analytical methods, fruit origin, and tissue source [14,16]. Structural elucidation based on methylation analysis, nuclear magnetic resonance spectroscopy, and chromatographic techniques has shown that polysaccharide fractions isolated from *L. ruthenicum* fruits include both neutral and acidic polysaccharides or glycoprotein-like fractions [51,53].

Several neutral glycoprotein fractions, such as LRGP1 and LRGP3, are mainly characterized by galactose- and arabinose-rich structures. These fractions commonly contain backbones composed of beta-D-galactopyranosyl residues, particularly (1→3)-linked beta-D-Galp units, with side chains attached mainly at the C-6 position. However, their branching patterns and side-chain compositions differ among fractions, indicating substantial structural diversity even within neutral polysaccharide or glycoprotein fractions. In contrast, acidic fractions such as LRGP5 contain higher proportions of uronic acids and exhibit pectin-like structural features. LRGP5 has been reported to contain a backbone composed of (1→4)-linked alpha-D-galacturonic acid and (1→2)-linked alpha-L-rhamnose residues, suggesting the presence of rhamnogalacturonan-type domains [52].

Further structural diversity has been observed in purified acidic polysaccharide fractions. For example, LRP3-S1 has been characterized as a highly branched pectic polysaccharide with a rhamnogalacturonan I-like backbone, whereas LRP1-S2 appears to contain arabinogalactan-type structural domains with glucuronic acid-containing side chains [48,53]. These findings suggest that *L. ruthenicum* polysaccharides are not a single homogeneous component but rather a group of structurally diverse macromolecules. Such diversity may partly explain the differences in solubility, viscosity, antioxidant capacity, immunomodulatory activity, and gut microbiota-regulating effects reported among different LRP fractions.

In addition to differences in monosaccharide composition and glycosidic linkages, some LRP fractions contain structural modifications, including acetylation and methyl esterification of uronic acid residues. These modifications may influence the charge distribution, conformation, water-holding capacity, and interaction with biological targets. Therefore, the biological activities of LRPs are likely determined by multiple structural parameters, including molecular weight, branching degree, uronic acid content, glycosidic linkage type, and chemical substitutions. Further studies are needed to clarify the precise structure-activity relationships of LRPs and to determine whether targeted structural modification can enhance their functional properties for food and nutraceutical applications.

For polysaccharides, structural characterization based on methylation analysis, NMR spectroscopy, monosaccharide profiling, molecular weight determination, and chromatographic separation usually provides only partial structural information, especially for highly branched acidic or glycoprotein-like fractions. This is consistent with the “proposed” status of the structural models summarized in Table 4. Complete assignment of linkage positions, branching points, substitution sites, conformational features, and protein-polysaccharide associations remains technically challenging for heterogeneous polysaccharide mixtures. These analytical uncertainties limit the strength of current structure-activity relationship conclusions. Future studies should therefore apply harmonized extraction and purification protocols, report detailed analytical conditions, use multiple complementary structural methods, and clearly distinguish confirmed structures from tentative assignments. Such rigor is necessary before the biological effects of *L. ruthenicum* and polysaccharides can be compared across studies or translated into standardized functional ingredients.

### 3.4. Lipids and Fatty Acids: Potential of L. ruthenicum Seed Oil

The seeds of *L. ruthenicum*, which are often generated as by-products during juice or extract processing, represent a potentially valuable source of edible oil. Previous studies have shown that *L. ruthenicum* seed oil contains a high proportion of unsaturated fatty acids, suggesting its potential application in functional foods, nutritional supplements, and cosmetic formulations [15,54]. Therefore, the valorization of *L. ruthenicum* seeds may contribute to the comprehensive utilization of this fruit and reduce processing waste.

#### 3.4.1. Fatty Acid Profile

The fatty acid profiles of L. ruthenicum oils from different materials are characterized by a predominance of unsaturated fatty acids, which account for more than 80% of the total fatty acids. Linoleic acid (C18:2*n*-6) is the major fatty acid, with reported relative contents ranging from approximately 59.4% to 74.6%, depending on the extraction method, sample origin, and analytical conditions [15,23]. This indicates that *L. ruthenicum* seed oil is a rich source of omega-6 polyunsaturated fatty acids.

Oleic acid (C18:1*n*-9) is another important unsaturated fatty acid in the oil, accounting for approximately 11.8% to 20.9% of total fatty acids [15,23]. In addition, gamma-linolenic acid (C18:3*n*-6), a biologically relevant omega-6 polyunsaturated fatty acid, has been detected at around 6.60% in seed oil [15]. Linolenic acid (C18:3*n*-3) has also been reported at approximately 2.98% in whole-fruit oil, suggesting that *L. ruthenicum* lipids may contain both omega-6 and omega-3 fatty acids, although their relative abundance differs among oil sources and extraction methods [23].

Saturated fatty acids are present at relatively lower levels. Palmitic acid (C16:0) is the predominant saturated fatty acid, representing approximately 4.9% to 7.1% of total fatty acids, whereas stearic acid (C18:0) accounts for about 1.4% to 5.3% [15,23]. Overall, the high proportion of unsaturated fatty acids, particularly linoleic acid and oleic acid, indicates that *L. ruthenicum* oil has a nutritionally favorable lipid profile. However, further studies are needed to evaluate its oxidative stability, refining characteristics, safety, sensory properties, and suitability for large-scale food or cosmetic applications.The main fatty acid components of *L. ruthenicum* seed oil are summarized in Table 5.

**Table 5:** Main fatty acid components in Lycium ruthenicum oils from different materials.

Category	Structural Formula	Extraction and Isolation Method	Content (Seed Oil)	Content (Dried-Fruit Oil)	References
Linoleic acid	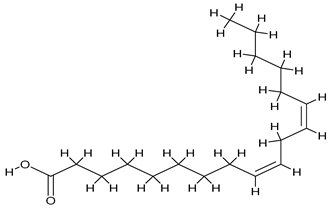	Soxhlet extraction (petroleum ether) or supercritical CO_2_ extraction; GC-MS analysis	74.58%	59.38%	Yossa Nzewua et al., 2020; Zhao et al., 2018 [15,23]
Oleic acid	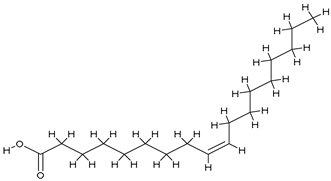	Soxhlet extraction (petroleum ether) or supercritical CO_2_ extraction; GC-MS analysis	11.82%	20.85%	Yossa Nzewua et al., 2020; Zhao et al., 2018 [15,23]
Palmitic acid	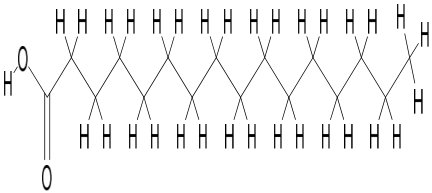	Soxhlet extraction (petroleum ether) or supercritical CO_2_ extraction; GC-MS analysis	4.88%	7.07%	Yossa Nzewua et al., 2020; Zhao et al., 2018 [15,23]
Linolenic acid	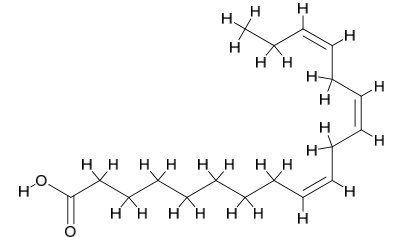	Soxhlet extraction (petroleum ether); GC-MS analysis	/	2.98%	Yossa Nzewua et al., 2020 [23]
Stearic acid	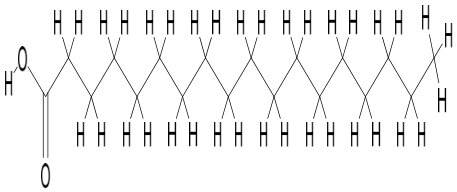	Soxhlet extraction (petroleum ether) or supercritical CO_2_ extraction; GC-MS analysis	1.40%	5.31%	Yossa Nzewua et al., 2020; Zhao et al., 2018 [15,23]
γ-Linolenic acid	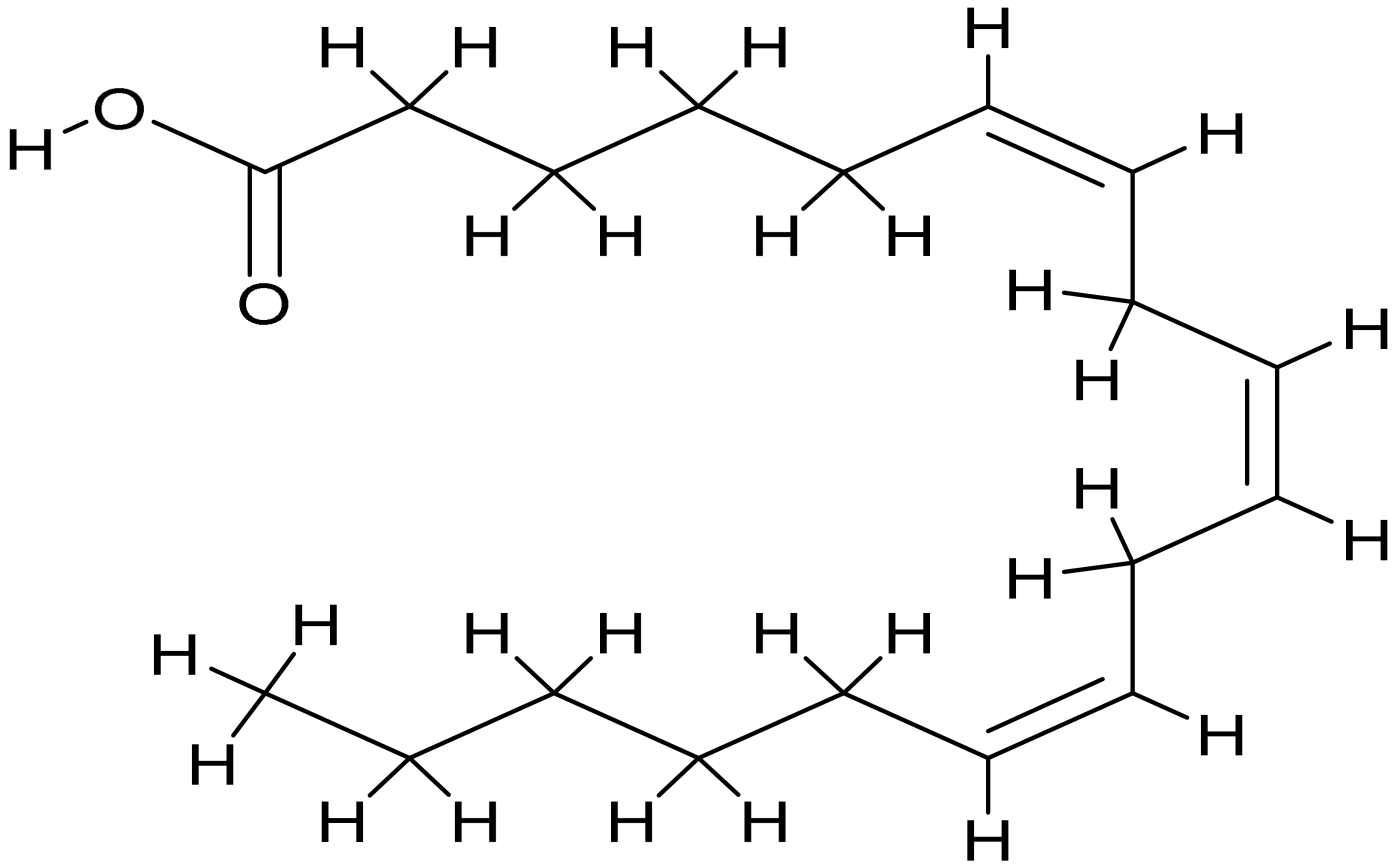	supercritical CO_2_ extraction; GC-MS analysis	6.60%	/	Zhao et al., 2018 [15]

#### 3.4.2. Lipid-Soluble Bioactives

In addition to fatty acids, *L. ruthenicum* seed oil contains several lipid-soluble bioactive compounds, including tocopherols and phytosterols. Tocopherols, mainly alpha-, gamma-, and delta-tocopherol, are important natural antioxidants in plant oils and may contribute to the oxidative stability of *L. ruthenicum* seed oil during storage and processing [15]. By inhibiting lipid peroxidation, these compounds may help protect unsaturated fatty acids from oxidative degradation and thereby maintain the nutritional quality of the oil [15,23].

Phytosterols, particularly beta-sitosterol, are another important class of lipid-soluble constituents reported in *L. ruthenicum* seed oil [15]. Owing to their structural similarity to cholesterol, phytosterols may interfere with intestinal cholesterol absorption and have therefore attracted attention for their potential role in lipid metabolism regulation [55]. In addition, tocopherols and phytosterols have been associated with antioxidant and anti-inflammatory effects, including modulation of oxidative stress- and inflammation-related pathways such as NF-kB signaling [23,55]. However, most of these functional interpretations are based on general knowledge of tocopherols and phytosterols or on in vitro and preclinical evidence; therefore, their specific contribution to the health effects of *L. ruthenicum* seed oil requires further confirmation.

The coexistence of lipid-soluble antioxidants, unsaturated fatty acids, and hydrophilic bioactive compounds such as anthocyanins and polysaccharides suggests that *L. ruthenicum* may act as a multi-component functional food matrix. Such a matrix may provide complementary antioxidant protection and contribute to its potential cardiometabolic benefits [15,23,55]. Nevertheless, the bioavailability, metabolic fate, dose–response relationships, and long-term safety of these lipid-soluble constituents remain insufficiently characterized. Further studies are therefore needed to determine whether the observed benefits of *L. ruthenicum* are attributable to individual compounds, combined phytochemical interactions, or the whole-fruit matrix.

### 3.5. Alkaloids and Other Nitrogenous Compounds

*L. ruthenicum* also contains a range of nitrogen-containing compounds, including betaine, polyamine derivatives, and amide-type constituents. Although these compounds are generally present at lower levels than anthocyanins, polysaccharides, and fatty acids, they may contribute to stress adaptation, nutritional value, and biological activities of the plant [56].

Betaine is one of the representative nitrogenous compounds reported in *Lycium* species. In plants, betaine functions as an osmoprotectant and may contribute to tolerance against salinity, drought, and other environmental stresses [57]. From a nutritional perspective, betaine can also serve as a methyl donor in one-carbon metabolism and has been associated with hepatic and metabolic functions [57]. Its presence in *L. ruthenicum* may therefore be relevant to both plant stress physiology and potential dietary value.

Beyond this, amide-type compounds, sometimes referred to as lycium amides, have been reported in *L. ruthenicum* and related *Lycium* species [58,59]. Preliminary studies suggest that some of these compounds may possess neuroprotective or other bioactive properties, but current evidence remains limited. In particular, their abundance in edible fruit tissues, absorption characteristics, mechanisms of action, and contribution to the overall health effects of *L. ruthenicum* require further investigation. Therefore, nitrogenous compounds should be regarded as promising but still underexplored constituents within the broader phytochemical profile of *L. ruthenicum*.

### 3.6. Mineral Profile and Trace Elements

The mineral composition of *L. ruthenicum* is closely related to its saline-alkaline and arid growth environments. Previous studies have shown that the fruit contains abundant macroelements, particularly potassium (K), sodium (Na), magnesium (Mg), and calcium (Ca), as well as trace elements such as iron (Fe), zinc (Zn), manganese (Mn), and selenium (Se) [23,60]. Among these elements, potassium has been reported at relatively high levels, with values reaching up to 17,631 mg/kg in some samples [23,61]. The high contents of K and Na may reflect the halophytic characteristics of *L. ruthenicum* and its adaptation to saline habitats.

Trace elements may contribute to the nutritional value of *L. ruthenicum*, as several of them are involved in antioxidant defense, enzyme activity, oxygen transport, and immune regulation. For example, Fe is essential for oxygen transport and hematopoiesis, Zn participates in immune and enzymatic functions, and Mn is associated with antioxidant enzyme systems. However, the mineral composition of *L. ruthenicum* may vary considerably according to geographical origin, soil mineral status, irrigation conditions, maturity stage, and postharvest processing.

In addition to beneficial minerals, the potential accumulation of toxic elements should be carefully considered. Because *L. ruthenicum* is grown in natural or semi-arid environments where soil composition varies substantially, elements such as lead (Pb), cadmium (Cd), arsenic (As), and mercury (Hg) should be routinely monitored [62,63]. This is particularly important for products intended for direct consumption, functional foods, nutraceutical preparations, and export markets. Therefore, mineral analysis should not only be used to evaluate nutritional quality but also to support quality control, geographical traceability, and food safety assessment.

### 3.7. Summary of Nutritional and Phytochemical Composition

In summary, the nutritional and phytochemical profile of *L. ruthenicum* is characterized by the coexistence of anthocyanins, non-anthocyanin polyphenols, polysaccharides, fatty acids, lipid-soluble bioactives, nitrogenous compounds, minerals, and trace elements. Among these constituents, acylated petunidin-derived anthocyanins represent the most distinctive compounds and largely account for the dark purple-black color and strong antioxidant potential of the fruit. In addition, phenolic acids, flavonols, and polyphenolic glycosides contribute to the total phenolic content, whereas LRPs provide structurally diverse water-soluble macromolecules with potential immunomodulatory and gut microbiota-regulating properties.

Taken as a whole, this compositional evidence points to a chemically rich food matrix, but it also highlights a practical question: which compounds remain stable, bioavailable, and biologically active after processing and digestion? Future work should place standardized compositional analysis alongside bioavailability testing, structure-activity evaluation, and quality-control studies so that individual compounds and the whole-fruit matrix can be interpreted together.

This compositional section also sets up the central biological question of the review. If anthocyanins, polysaccharides, lipids, and nitrogenous compounds are all present, which mechanisms can reasonably explain the reported health-related effects, and where does the current evidence remain too preliminary for strong claims?

## 4. Biological Activities and Health-Related Effects of *L. ruthenicum*

*L. ruthenicum* has long been used as a medicinal and edible plant in traditional dietary and ethnomedicinal systems. Its traditional applications, including nourishing effects, visual health support, and general health maintenance, have encouraged modern studies on its biological activities. Evidence from chemical assays, cell models, animal experiments, and limited applied studies suggests that *L. ruthenicum* and its bioactive constituents may exert antioxidant, anti-inflammatory, neuroprotective, metabolic-regulatory, anti-fatigue, chemopreventive-related, and immunomodulatory effects.

Across these studies, a recurring pattern is that biological activity is usually linked to more than one class of constituents. Anthocyanins, polysaccharides, phenolic acids, flavonoids, fatty acids, and lipid-soluble antioxidants may act through partially overlapping mechanisms. At the same time, most evidence remains preclinical, and many studies use crude extracts whose composition is not fully standardized. The following discussion therefore emphasizes possible mechanisms while keeping the strength of evidence in view.

### 4.1. Antioxidants

Oxidative stress is closely associated with cellular injury, aging, metabolic disorders, neurodegeneration, inflammation, and other pathological processes. The antioxidant activity of *L. ruthenicum* is therefore considered one of the major mechanisms underlying its potential health-promoting effects. Available studies suggest that *L. ruthenicum* may exert antioxidant activity through two complementary mechanisms: direct scavenging of reactive oxygen species and modulation of endogenous antioxidant defense pathways.

#### 4.1.1. Direct Radical-Scavenging Capacity

Extracts of *L. ruthenicum* exhibit strong radical-scavenging activity, which is largely attributed to their high content of anthocyanins, especially petunidin-derived compounds. These compounds can donate electrons or hydrogen atoms to neutralize free radicals and reduce oxidative reactions. In commonly used antioxidant assays, including DPPH, ABTS, and FRAP assays, antioxidant capacity has been reported to correlate positively with total anthocyanin content, suggesting that anthocyanins are major contributors to the antioxidant potential of *L. ruthenicum* (Table 6) [39].

The structural features of *L. ruthenicum* anthocyanins may further shape their antioxidant relevance. Glycosylation and acylation can improve pigment stability and may help preserve antioxidant activity under changing food-processing conditions, including variations in pH, temperature, and light exposure [64,65]. Non-anthocyanin phenolics, such as chlorogenic acid and caffeic acid, together with other antioxidant constituents, add to the radical-scavenging profile of the fruit [66]. Whether these compounds act additively or synergistically remains an open experimental question.

**Table 6:** Preclinical Biological Activities and Health-Relevant Effects of *Lycium ruthenicum* and Its Bioactive Constituents.

Biological Activity	Studied Material	Chemical Characterization	Model	Dose/Concentration	Main Finding	Proposed Mechanism	Key Limitation	Evidence Tier	Refs.
Antioxidant Activity	*Purified* *anthocyanin (97%) + crude extract , L. ruthenicum*	Anthocyanins (especially petunidin-derived)	In vitro chemical assays (DPPH, ABTS, FRAP)	25–500 µg/mL	Antioxidant capacity ↑ with TAC	Direct radical scavenging	In vitro only; synergy unclear	In vitro	[39,65,66]
	*L. ruthenicum* polyphenols	Nine phenolic compounds	Acrylamide-induced liver injury in male SD rats	50, 100, 200 mg/kg/d, i.g., 19 d	Alleviated hepatic oxidative damage	Nrf2/HO-1 activation	Preclinical only; human relevance unclear	In vivo (SD rats)	[67]
	*L. ruthenicum* extract	TAC: 43.64 ± 9.28 Pt eq. g/100 g DW	Ethanol-induced acute ALD in *C57BL/6J* male mice	50, 125, 375 mg/kg/d, i.g., 21 d	Relieved oxidative injury	Nrf2/HO-1 activation; NF-κB inhibition	Preclinical only; human relevance unclear	In vivo (*C57BL/6J* mice)	[68]
	*L. ruthenicum* anthocyanins	Anthocyanins (purity: 87.54%)	D-galactose-induced aging SD female rats	100 mg/kg/d, i.g., 8 wks	Rebuilds intrinsic antioxidant system	Lower AGE/MDA; higher GSH, MT, GSH-Px, CAT, T-SOD	Preclinical only; human relevance unclear	In vivo (SD rats)	[69]
	*L. ruthenicum* anthocyanin extract	30 compounds identified by UHPLC-MS/MS	CdCl_2_-induced testicular toxicity in male ICR mice	50, 250, 500 mg/kg/d, i.g., 28 d	Enhanced antioxidant defenses	SIRT1/Nrf2/Keap1 modulation	Preclinical only; human relevance unclear	In vivo (ICR mice)	[70]
Neuroprotective and Cognitive-Enhancing Effects	LRP3	LRP3	Neonatal rat primary cortical neurons	5, 10, 20 µM	Improved cell viability; reduced neuronal apoptosis	↑ Bcl-2, ↓ Bax, ↓ cleaved caspase-3; ↓ ROS; Nrf2 signaling	PK, BBB, dose, translation unclear	In vitro (Neonatal rat primary cortical neurons)	[47]
	new polyphenolic glycosides: lyciumerin A–I	nine new polyphenolic glycosides and 16 known compounds	6-hydroxydopamine-induced PC12 cell injury model	25, 50, and 100 µM	Improved viability	Selective MAO-B inhibition	Absorption, BBB and in vivo activity unknown	In vitro (PC12 cells)	[42,71]
	*L. ruthenicum* extract	Anthocyanins and spermidines	D-Gal-induced aging Kunming mice	25, 50, 100 mg/kg/d, i.g., 6 wks	Improved learning and memory	↓ MDA; ↑ SOD, GSH-Px	Human studies required	In vivo (Kunming mice)	[72]
Metabolic-Regulatory Effects	*L. ruthenicum* extract	flavonoids extracts	WD-induced NAFLD ApoE^−^/^−^ mice (*C57BL/6*)	140 mg/kg/d, i.g., 4 wks	Attenuated hepatic steatosis; ↓ TG and TC	↓ De novo lipogenesis genes	Human data insufficient	In vivo (*C57BL/6* mice)	[61,73]
	*L. ruthenicum* anthocyanins	12 anthocyanins identified by UPLC-ESI-MS/MS	HFD-induced insulin resistance in *C57BL/6J* mice	50, 100, 200 mg/kg/d, i.g., 12 wks	Reduced postprandial glucose spikes in OGTT	Amelioration of hepatic inflammation and oxidative stress	Human intervention required	In vivo (*C57BL/6J* mice)	[74]
	*L. ruthenicum* anthocyanins	14 anthocyanins identified by HPLC-MS	Caco-2 cell	5, 10, 20, 40, 80 µg/mL	Delayed carbohydrate digestion; ↓ postprandial glucose	Inhibited alpha-glucosidase activity	Human intervention required	In vitro (Caco-2 cells)	[75]
	*L. ruthenicum* anthocyanins	14 anthocyanins identified by HPLC-MS	Insulin-resistant Hep-G2 cells	25 µg/mL	Improved glucose consumption and glucose uptake	PI3K/Akt activation	Human intervention required	In vitro (Hep-G2 cells)	[75]
Cardioprotective	*L. ruthenicum* extract	Anthocyanin content: 24.1 ± 7.7 mg/g	Exhaustive exercise-induced cardiac injury in SD rats	10 and 30 mg/rat/d, i.g., 7 d	Ameliorated myocardial damage	↓ Serum CK-MB	Cardiovascular effects insufficiently characterized	In vivo (SD rats)	[76]
	*L. ruthenicum* aqueous extract	/	ISO-induced acute myocardial ischemia in ICR mice	375 and 750 mg/kg/d, i.g., 8 d	Improved cardiac morphology	↓ CK-MB, LDH, MDA	Cardiovascular effects insufficiently characterized	In vivo (ICR mice)	[23]
Immunomodulatory Effects and Gut Health	*Crude anthocyanins or P3G, LRM*	P3G and ACN	DSS-induced colitis in *C57BL/6* mice	200 mg/kg/d, i.g., days 9–16	Alleviated colitis symptoms; reduced TNF-α, IL-6, IL-1β, IFN-γ	NF-κB modulation	Targets, dose–response unclear	In vivo (*C57BL/6* mice)	[77]
	*Crude anthocyanins + P3G, LRM*	P3G and ACN	DSS-induced colitis in *C57BL/6* mice	200 mg/kg/d, i.g., days 9–16	Improves gut microbial dysbiosis	Microbiota-SCFA-immune axis	Direct or indirect effects unclear	In vivo (*C57BL/6* mice)	[77,78,79,80]
	*LRWP*	Total sugar: 95.37%; anthocyanin: 5.53 mg CGE/g	MDCK cells/influenza A/H3N2	15.6–125 µg/mL	Inhibited influenza A/H3N2 virus replication	Interference with viral replication/post-entry	Cell-level only; mechanism unclear	In vitro (MDCK cells)	[81]
Antiproliferative and chemopreventive effects	Pt3G	Pt3G	DU145 human prostate cancer cells	100, 200, 400 µg/mL, in vitro, 24 h	Inhibit cell proliferation and induce apoptosis	ROS ↑; caspase-3 activation; ↑ PTEN; PI3K/Akt inhibition	Selectivity, bioavailability, in vivo data lacking	In vitro (DU145 cells)	[82,83]
	*L. ruthenicum* polyphenols	Nine phenolic compounds	ACR-induced liver injury in SD rats	50, 100, 200 mg/kg/d, i.g., 19 d	Preserved mitochondria; ↓ oxidative damage	Nrf2 activation	Context-dependent redox regulation	In vivo (SD rats)	[67]
Anti-fatigue	*Fruit extracts of Lycium ruthenicum*	/	EIF in Kunming mice	0.05, 0.1, 0.2, 0.5 mg/g, i.g., 28 d	Improvement in endurance	BUN, LDH; modulation of specific microRNAs	Preclinical only; human relevance unknown	In vivo (Kunming mice)	[84,85,86]

↑ indicates an increase; ↓ indicates a decrease.

#### 4.1.2. Modulation of Endogenous Antioxidant Signaling

Beyond direct radical scavenging, *L. ruthenicum* constituents may also enhance cellular antioxidant defense by regulating endogenous signaling pathways. Among these pathways, the nuclear factor erythroid 2-related factor 2/antioxidant response element pathway, commonly referred to as the Nrf2/ARE pathway, has received particular attention [87]. Under oxidative stress, Nrf2 translocates from the cytoplasm to the nucleus and binds to ARE sequences, thereby promoting the transcription of cytoprotective genes, including heme oxygenase-1, superoxide dismutase, catalase, and glutathione peroxidase.

Several studies have reported that *L. ruthenicum* extracts, anthocyanin-rich fractions, and LRPs may regulate oxidative stress-related pathways. In acrylamide-induced liver injury models, *L. ruthenicum* polyphenols restored antioxidant enzyme activity and alleviated hepatic oxidative damage by activating the Nrf2/HO-1 pathway (Figure 2 and Table 6) [67]. In ethanol-induced liver injury models, *L. ruthenicum* extracts relieved oxidative injury through dual regulation of the Nrf2/HO-1 antioxidant pathway and NF-κB inflammatory pathway [68]. In D-galactose-triggered aged liver rat models, LRA cut serum AGE and MDA levels while raising endogenous antioxidants including GSH, metallothionein and key antioxidant enzymes (GSH-Px, CAT, T-SOD). It rebuilds the body’s intrinsic antioxidant system and eases age-linked liver oxidative damage by correcting disrupted amino acid metabolism (Table 6) [69]. In addition, anthocyanins from *L. ruthenicum* have been reported to alleviate cadmium-induced testicular toxicity by modulating the SIRT1/Nrf2/Keap1 pathway, thereby enhancing antioxidant defenses and reducing oxidative injury (Table 6) [70]. These findings are consistent with broader evidence that dietary phytochemicals can regulate Nrf2-related signaling and protect against oxidative stress-associated damage [88].

Taken together, current evidence indicates that the antioxidant activity of *L. ruthenicum* reflects both chemical radical-scavenging reactions and biological regulation of endogenous defense systems. The mechanistic picture is promising, but many studies remain limited to cells and animals. Clarifying which constituents activate Nrf2, how well they are absorbed, what concentrations are physiologically achievable, and whether tissue-specific effects occur in humans will be essential for evaluating dietary relevance.

### 4.2. Neuroprotective and Cognitive-Improvement-Related Effects

The neuroprotective potential of *L. ruthenicum* has attracted attention because oxidative stress, neuroinflammation, mitochondrial dysfunction, and apoptosis are shared features of ischemic injury, neurodegenerative disorders, and aging-related cognitive decline. Current evidence suggests that polysaccharides, anthocyanins, and polyphenolic glycosides may protect neural cells by attenuating oxidative stress, reducing neuronal apoptosis, regulating inflammatory signaling, and affecting neurotransmitter-related pathways. Since most studies are based on cellular or animal models, the cognitive and neurological relevance in humans remains to be tested.

#### 4.2.1. Mitigation of Ischemic Injury

Cerebral ischemia–reperfusion injury involves a cascade of pathological events, including excitotoxicity, oxidative stress, mitochondrial dysfunction, inflammatory responses, and apoptosis. LRP3, a purified polysaccharide fraction isolated from *L. ruthenicum*, has been reported to exert protective effects in oxygen-glucose deprivation/reperfusion-induced injury models of neonatal rat primary cortical neurons (Table 6) [47]. In this model, LRP3 pretreatment significantly improved cell viability and reduced neuronal apoptosis.

LRP3 appears to regulate apoptosis-related proteins by increasing anti-apoptotic Bcl-2 and decreasing pro-apoptotic Bax (Figure 3). It also reduces cleaved caspase-3, indicating inhibition of caspase-dependent apoptosis [47]. The same experimental context links this effect to lower ROS generation and activation of antioxidant defense pathways, including Nrf2-related signaling. These data suggest a combined anti-apoptotic and antioxidant mechanism, although in vivo pharmacokinetics, blood–brain barrier permeability, effective dose, and translational relevance still need clarification.

#### 4.2.2. Protection Against Neurotoxicity and Neurodegeneration

Several studies have also investigated the effects of *L. ruthenicum* constituents in neurotoxicity models related to neurodegenerative disorders. Monoamine oxidase B (MAO-B) is involved in dopamine metabolism and can contribute to the generation of oxidative by-products. Therefore, MAO-B inhibition is considered a potential strategy for reducing oxidative stress associated with dopaminergic neuronal injury.

Polyphenolic glycosides isolated from *L. ruthenicum*, including lyciumserin-type compounds, have been reported to exhibit selective MAO-B inhibitory activity (Figure 3 and Table 6) [42,71]. By inhibiting MAO-B, these compounds may help reduce dopamine degradation and oxidative by-product formation, which is relevant to Parkinson disease-related mechanisms. In a 6-hydroxydopamine-induced PC12 cell injury model, several polyphenolic glycosides improved cell viability and showed relatively low cytotoxicity. For example, lyciumserin A and lyciumserin B increased cell survival in this model, with protective effects reported to be comparable to those of the positive control rasagiline under the same experimental conditions [42].

These results place non-anthocyanin polyphenolic glycosides within the broader neuroprotective profile of *L. ruthenicum*. The evidence is still early, being based mainly on enzyme inhibition assays and cell models. Further work should determine whether these compounds are absorbed, whether they cross the blood–brain barrier, and whether the observed activity persists in animal or human settings relevant to neurodegenerative disease prevention.

#### 4.2.3. Anti-Aging and Cognitive-Improvement-Related Effects

Aging-related cognitive decline is closely associated with oxidative stress, neuroinflammation, neuronal loss, and metabolic dysregulation. In D-galactose-induced aging mouse models, ethanolic extracts of *L. ruthenicum* have been reported to improve learning and memory performance, as indicated by behavioral tests such as the Morris water maze [72]. Treated mice showed reduced escape latency and increased time spent in the target quadrant, suggesting improvement in spatial learning and memory.

At the biochemical level, *L. ruthenicum* extract reduced malondialdehyde accumulation in brain tissue and increased the activities of antioxidant enzymes, including SOD and GSH-Px (Table 6) [72]. These results suggest that *L. ruthenicum* may help maintain redox homeostasis and reduce oxidative damage in aging-related brain injury models. In addition, petunidin-3,5-*O*-diglucoside, a major anthocyanin-related compound, has been reported to alleviate cognitive deficits, reduce neuronal loss, suppress neuroinflammation, and restore hippocampal metabolic profiles, possibly through regulation of amino acid metabolism and antioxidant defenses (Figure 3) [89].

The evidence from aging-related models suggests that *L. ruthenicum* may influence cognition through coordinated regulation of oxidative stress, inflammatory signaling, neuronal apoptosis, and metabolic homeostasis [90]. This interpretation is supported by several converging markers rather than by a single endpoint. Even so, well-designed human studies are required before these preclinical findings can be translated into dietary recommendations or functional food claims.

### 4.3. Metabolic-Regulatory Effects

Metabolic syndrome is characterized by a cluster of metabolic abnormalities, including obesity, dyslipidemia, insulin resistance, hyperglycemia, and hypertension. In studies of *L. ruthenicum*, metabolic regulation is usually discussed through changes in lipid accumulation, glucose homeostasis, oxidative stress, and inflammation. The relevant evidence comes mainly from anthocyanins, polysaccharides, phenolic compounds, and lipid-soluble constituents, yet human data remain insufficient.

#### 4.3.1. Regulation of Hepatic Lipid Metabolism and NAFLD-Related Injury

Non-alcoholic fatty liver disease is closely associated with metabolic syndrome and is characterized by excessive lipid accumulation in hepatocytes, oxidative stress, inflammation, and impaired lipid metabolism. In mice fed a cholesterol-enriched Western diet, supplementation with *L. ruthenicum* extract was reported to attenuate hepatic steatosis and reduce hepatic triglyceride and total cholesterol accumulation (Table 6) [61]. These findings suggest that *L. ruthenicum* may have protective effects against diet-induced hepatic lipid dysregulation.

The role of *L. ruthenicum* in hepatic lipid metabolism may involve lipid synthesis, lipid transport, and fatty acid remodeling rather than one linear pathway. Transcriptomic analysis showed that genes associated with de novo lipogenesis, including peroxisome proliferator-activated receptor gamma and fatty acid synthase, were downregulated after intervention [73]. Such changes provide a plausible explanation for reduced hepatic lipid deposition, while the specific role of stearoyl-CoA desaturase-1 should be interpreted carefully because it is more closely related to fatty acid desaturation and lipid composition than to fatty acid oxidation itself.

#### 4.3.2. Hypoglycemic Effects and Insulin Sensitivity

*L. ruthenicum* anthocyanins have shown promise in regulating glucose metabolism and improving insulin sensitivity. In high-fat diet-induced *C57BL/6J* mice, oral administration of these anthocyanins effectively reduced postprandial glucose spikes in oral glucose tolerance tests (OGTT) (Table 6) [74]. Mechanistic studies suggest this effect is primarily achieved through the amelioration of hepatic inflammation and oxidative stress, and the subsequent inhibition of hepatic gluconeogenesis [74]. In parallel, in vitro evidence indicates that these anthocyanins may also act at the intestinal level. One possible mechanism is the inhibition of alpha-glucosidase, an intestinal enzyme involved in the hydrolysis of carbohydrates into absorbable glucose [73]. In Caco-2 cell-based models, *L. ruthenicum* anthocyanins were reported to inhibit alpha-glucosidase activity, suggesting a potential ability to delay carbohydrate digestion and reduce postprandial glucose availability (Table 6) [75].

Beyond digestive enzyme inhibition, *L. ruthenicum* anthocyanins may also improve insulin resistance through intracellular signaling pathways. In insulin-resistant HepG2 cells induced by high glucose and palmitic acid, treatment with *L. ruthenicum* anthocyanins improved glucose consumption and glucose uptake (Table 6) [75]. Mechanistically, these effects were associated with activation of the PI3K/Akt signaling pathway, as indicated by increased phosphorylation of IRS-2, Akt, and GSK3beta. Activation of this pathway may promote glycogen synthesis and improve cellular glucose utilization (Figure 4).

Mitochondrial protection provides another link between glucose metabolism and oxidative stress. *L. ruthenicum* anthocyanins were reported to reduce cellular and mitochondrial ROS, restore ATP production, and recover mitochondrial membrane potential [75]. These findings suggest that glucose-regulatory effects may involve digestive enzyme inhibition, insulin signaling, oxidative stress reduction, and mitochondrial function. Validation in animal models and human intervention studies is still required.

#### 4.3.3. Cardioprotective Potential

*L. ruthenicum* may also exert cardioprotective effects, mainly through antioxidant and lipid metabolism-related mechanisms. Its seed oil contains a high proportion of unsaturated fatty acids, particularly linoleic acid and oleic acid, as well as phytosterols and tocopherols, which may contribute to the maintenance of lipid nutritional quality and cardiovascular-related health potential [15]. However, the specific cardiovascular effects of *L. ruthenicum* seed oil remain insufficiently characterized and require further investigation.

The cardioprotective effects of *L. ruthenicum* have been evaluated in multiple experimental models. In rats subjected to exhaustive exercise, supplementation with *L. ruthenicum* extract significantly reduced serum creatine kinase-MB (CK-MB) levels and ameliorated myocardial histopathological damage (Table 6) [76]. Similarly, in isoproterenol-induced acute myocardial ischemia in mice, pretreatment with *L. ruthenicum* aqueous extract decreased serum CK-MB and lactate dehydrogenase (LDH) activities, reduced myocardial malondialdehyde (MDA) content, and improved cardiac tissue morphology (Table 6) [23].

### 4.4. Immunomodulatory Effects and Gut Health

The interaction among dietary bioactive compounds, host immune responses, and gut microbiota has become a central theme in *L. ruthenicum* research. Anthocyanins and polysaccharides appear to regulate inflammatory responses, intestinal barrier-related functions, and microbial composition. These effects are especially relevant to intestinal inflammation and metabolic health, although current evidence is still dominated by animal models and in vitro assays.

#### 4.4.1. Immunomodulatory and Anti-Inflammatory Effects

The immunomodulatory effects of *L. ruthenicum* have been investigated mainly in inflammation-related experimental models. In dextran sulfate sodium-induced colitis mice, oral administration of *L. ruthenicum* anthocyanins alleviated colitis-associated symptoms, including body weight loss, colon shortening, and colonic tissue injury (Table 6) [77]. These protective effects were accompanied by reduced levels of pro-inflammatory cytokines, including TNF-alpha, IL-6, IL-1beta, and IFN-gamma.

At the molecular level, *L. ruthenicum* anthocyanins also decreased the expression of inflammatory mediators such as inducible nitric oxide synthase, cyclooxygenase-2, and monocyte chemoattractant protein-1 [77]. These changes suggest that *L. ruthenicum* may attenuate intestinal inflammation partly by modulating NF-kB-related inflammatory signaling [91,92]. Nevertheless, the precise upstream targets, dose–response relationships, and contribution of individual anthocyanin compounds remain to be further clarified.

#### 4.4.2. Modulation of Gut Microbiota and Short-Chain Fatty Acids

In addition to regulating inflammatory mediators, *L. ruthenicum* anthocyanins may influence intestinal health by modulating the gut microbiota. In DSS-induced colitis models, anthocyanin treatment was reported to improve gut microbial dysbiosis, increasing the relative abundance of potentially beneficial bacteria, such as Akkermansia, Faecalibacterium, and Lactobacillus, while reducing the abundance of harmful or inflammation-associated bacterial populations (Figure 5 and Table 6) [77,78,79].

These microbial changes were associated with increased levels of short-chain fatty acids, including acetate, propionate, and butyrate, in cecal and fecal samples [77,79,80]. SCFAs, especially butyrate, are important microbial metabolites involved in maintaining intestinal epithelial integrity, regulating immune responses, and suppressing excessive inflammation. Therefore, the gut-protective effects of *L. ruthenicum* anthocyanins may be partly mediated by the microbiota-SCFA-immune regulation axis. However, it remains unclear whether these microbial changes are direct effects of anthocyanins, consequences of improved intestinal inflammation, or the result of anthocyanin-derived microbial metabolites.

#### 4.4.3. Antiviral Activity

Preliminary evidence also suggests that *L. ruthenicum* may possess antiviral activity. In vitro studies have shown that *L. ruthenicum* fruit extract inhibited influenza A/H3N2 virus replication, with a reported half-maximal inhibitory concentration of 17.58 microgram/mL (Figure 5 and Table 6) [81]. Within the tested concentration range of 15.6–125 microgram/mL, the extract showed no significant cytotoxicity, suggesting an acceptable safety profile in the corresponding cell model [81].

Mechanistic observations indicate that the antiviral effect may not be primarily mediated by blocking viral adsorption, but may instead involve interference with viral replication or post-entry processes [81]. Although these in vitro findings provide preliminary support for *L. ruthenicum* as a natural antiviral resource, the existing evidence remains limited to cell-level tests. Key unresolved issues include the specific antiviral constituents, complete molecular mechanism, in vivo therapeutic efficacy, and functional performance under routine dietary intake. These questions need to be answered before respiratory health-related applications can be proposed with confidence.

### 4.5. Antiproliferative and Chemopreventive-Related Effects

The antiproliferative and chemopreventive-related effects of *L. ruthenicum* have been studied in selected cell and animal models. Anthocyanins and polyphenols may influence cancer cell survival, apoptosis-related signaling, oxidative stress, and toxicant-induced tissue injury. Because the evidence is preclinical, these observations should be interpreted as mechanistic leads rather than direct proof of cancer prevention or treatment in humans.

#### 4.5.1. Apoptosis Induction in Prostate Cancer Cells

Petunidin-3-*O*-glucoside, an anthocyanin monomer associated with *L. ruthenicum*, has been investigated in DU145 human prostate cancer cells. Petunidin-3-*O*-glucoside treatment was reported to increase intracellular reactive oxygen species levels, which may act as a signaling trigger for apoptosis rather than merely causing nonspecific oxidative injury [82,83]. Increased ROS production was associated with upregulation of the tumor suppressor PTEN and subsequent inhibition of the PI3K/Akt signaling pathway, a key pathway involved in cell survival and proliferation (Figure 6).

Inhibition of PI3K/Akt signaling further promoted caspase-3 activation and apoptosis in DU145 cells. In addition, treatment was associated with G1-phase cell cycle arrest, suggesting that it may suppress cancer cell proliferation through coordinated regulation of cell cycle progression and apoptosis-related pathways (Table 6) [82,83]. These findings indicate that petunidin-3-*O*-glucoside has potential antiproliferative activity in prostate cancer cell models. Nevertheless, further studies are required to confirm its selectivity, bioavailability, effective concentration, in vivo antitumor activity, and relevance under dietary exposure conditions.

#### 4.5.2. Protection Against Acrylamide-Induced Hepatotoxicity

The same redox-regulatory theme appears in toxicant-induced tissue injury models. *L. ruthenicum* polyphenols have been reported to protect normal tissues against acrylamide-induced oxidative injury. Acrylamide, a food-processing contaminant formed during high-temperature cooking, can induce hepatotoxicity partly through oxidative stress, mitochondrial dysfunction, and disruption of antioxidant defense systems. In acrylamide-induced liver injury models, *L. ruthenicum* polyphenols preserved mitochondrial structure and function and reduced oxidative damage [67].

Mechanistically, these protective effects were associated with activation of Nrf2-related antioxidant signaling. *L. ruthenicum* polyphenols upregulated downstream antioxidant and detoxification-related proteins, including HO-1, GCLC, GCLM, and NQO1, while reducing oxidative stress markers such as ROS and MDA (Figure 6 and Table 6) [67]. They also increased antioxidant defenses, including GSH and SOD, and helped restore mitochondrial ATPase activity and the expression of respiratory chain complex-related genes. These findings suggest that *L. ruthenicum* polyphenols may alleviate acrylamide-induced hepatotoxicity by enhancing antioxidant defense and improving mitochondrial energy metabolism.

This dual pattern of redox regulation is scientifically interesting: in some cancer cell models, *L. ruthenicum* constituents may promote ROS-mediated apoptosis, whereas in normal tissue injury models they may reduce excessive oxidative stress. The apparent context dependence requires careful interpretation and should be examined with more selective models, animal experiments, and eventually human-relevant exposure conditions.

### 4.6. Anti-Fatigue and Physical Performance-Related Effects

The anti-fatigue potential of *L. ruthenicum* has been evaluated mainly in animal exercise models. In exhaustive swimming tests, fruit extracts significantly prolonged swimming time in mice, suggesting a possible improvement in endurance (Table 6) [84,85,86]. These effects may relate to improved energy metabolism, reduced accumulation of fatigue-related metabolites, and attenuation of exercise-induced oxidative stress. The underlying mechanisms of the anti-fatigue effects of *L. ruthenicum* extracts are summarized in Figure 7.

Biochemical analyses showed that *L. ruthenicum* extracts reduced blood urea nitrogen and lactate dehydrogenase levels after exhaustive exercise [84,85]. Blood urea nitrogen is commonly used as an indicator of protein catabolism and fatigue-related metabolic stress, whereas lactate dehydrogenase is associated with tissue and muscle injury. In addition, *L. ruthenicum* treatment helped preserve glycogen stores in the liver and skeletal muscle, indicating improved energy reserve maintenance during prolonged physical activity.

Emerging molecular evidence further suggests that *L. ruthenicum* may regulate exercise-related metabolic pathways. High-throughput sequencing analyses have indicated that *L. ruthenicum* extracts may modulate specific microRNAs involved in energy metabolism, oxidative stress, and inflammatory responses [84,85]. These findings provide a possible molecular explanation for its anti-fatigue effects. However, current evidence remains limited to preclinical models, and further studies are required to determine the active constituents, dose–response relationships, safety, and relevance to human exercise performance.

### 4.7. Summary of Biological Activities and Health Benefits

Experimental models describe a broad spectrum of *L. ruthenicum* activities, including antioxidant, neuroprotective, metabolic-regulatory, immunomodulatory, gut microbiota-regulatory, hepatoprotective, antiproliferative, and anti-fatigue effects. Rather than representing isolated actions, these activities are linked to a shared pool of bioactive constituents, particularly anthocyanins, polysaccharides, phenolic acids, flavonoids, fatty acids, tocopherols, phytosterols, and nitrogenous compounds.

When the biological evidence is viewed across models, *L. ruthenicum* appears to act as a multi-component food matrix rather than as a source of a single active compound. Its reported activities involve Nrf2/ARE antioxidant signaling, NF-kB-mediated inflammatory signaling, PI3K/Akt insulin signaling, apoptosis-related pathways, lipid metabolism-related pathways, and the gut microbiota-SCFA axis. The next challenge is to connect these mechanisms with standardized products, realistic intake levels, and human outcomes.

## 5. Food Processing, Stability Enhancement, and Product Development

The transformation of *Lycium ruthenicum* from a traditionally consumed wild resource into a high-value functional food ingredient depends largely on appropriate processing and preservation technologies. Although *L. ruthenicum* is rich in anthocyanins, polyphenols, polysaccharides, fatty acids, and other bioactive constituents, many of these compounds are sensitive to processing and storage conditions. In particular, anthocyanins are susceptible to degradation caused by heat, light, oxygen, enzymes, and pH variation, which may lead to color fading, reduced antioxidant capacity, and decreased product quality. Therefore, improving extraction efficiency, enhancing anthocyanin stability, optimizing fermentation processes, and developing value-added products are central to the industrial utilization of *L. ruthenicum*. However, stable industrial production starts with raw-material consistency. Variations in fruit maturity, geographical origin, drying protocols, particle size, and contaminant levels cannot be fully compensated by downstream processing. Concentration-oriented procedures may enrich bioactive compounds together with undesirable contaminants such as heavy metals. Pre-processing steps including cleaning, drying, milling, and storage should minimize oxidation and enzymatic degradation to lay a foundation for subsequent extraction, fermentation, and formulation.

From a processing perspective, the key question is no longer simply whether *L. ruthenicum* contains abundant anthocyanins, but whether these compounds can be retained in a stable, acceptable, and safe product. Heat, light, oxygen, enzymes, and pH variation can all reduce pigment stability and product quality. The development of extraction, stabilization, fermentation, and packaging technologies therefore determines how much of the fruit’s chemical potential can be translated into commercial foods.

This processing-oriented perspective is important because many promising bioactivities are reported under controlled laboratory conditions. In actual food systems, the same compounds encounter heat, oxygen, enzymes, microbial metabolism, packaging materials, and storage conditions, all of which can reshape the final product.

### 5.1. Advanced Extraction Technologies

Efficient recovery of bioactive compounds is a decisive step in product development. Conventional solvent extraction, especially with acidified ethanol or methanol, remains useful for laboratory analysis but often involves long extraction times, high solvent consumption, and limited suitability for direct food applications. Green, food-grade, and high-efficiency extraction strategies have therefore become increasingly important.

#### 5.1.1. Beta-Cyclodextrin-Assisted Green Extraction

Beta-cyclodextrin-assisted extraction has been proposed as a promising green strategy for recovering anthocyanins from *L. ruthenicum*. Beta-cyclodextrin is a cyclic oligosaccharide with a hydrophobic internal cavity and a hydrophilic outer surface. Through host-guest interactions, beta-cyclodextrin can form inclusion complexes with hydrophobic regions of anthocyanin molecules, particularly aromatic and acylated moieties. This interaction may improve the apparent solubility, extraction efficiency, and stability of anthocyanins in aqueous systems [33].

Compared with conventional organic solvent extraction, beta-cyclodextrin-assisted aqueous extraction offers several potential advantages. At the level of raw material control, it reduces the use of organic solvents and is therefore more compatible with food-grade processing. Variation becomes more evident when the formation of inclusion complexes may protect anthocyanins from oxidation, thermal degradation, and structural transformation during extraction. A further complication arises because response surface methodology has been used to optimize extraction parameters, including beta-cyclodextrin concentration, extraction temperature, extraction time, and solid–liquid ratio, thereby improving anthocyanin yield and process reproducibility [33].

Analytical studies using UPLC-DAD show that beta-cyclodextrin-assisted extraction can achieve anthocyanin recovery comparable to conventional methanol-based methods while improving stability and downstream food compatibility [33]. Its industrial value will depend on cost, sensory influence, regulatory acceptability, and performance at pilot and production scales. Practical scale-up also needs to consider extraction-system viscosity, filtration performance, and residual β-cyclodextrin in final extracts. Whether cyclodextrin is retained or removed from products should be factored into process design, rather than evaluating this method solely based on laboratory-scale anthocyanin recovery [33].

#### 5.1.2. Chromatographic Purification and Quality Standardization

The anthocyanin profile of *L. ruthenicum* is chemically complex, consisting mainly of acylated petunidin, delphinidin, and malvidin derivatives. Many of these compounds exist as closely related structural isomers, including cis- and trans-configured acylated anthocyanins [28]. Because these isomers share similar molecular weights and hydrophobic properties, conventional reversed-phase chromatographic systems, such as C18 or C8 columns, may not fully resolve them [26].

Advanced chromatographic methods have therefore been developed to improve the separation and identification of *L. ruthenicum* anthocyanins. Mixed-mode stationary phases, such as reversed-phase/strong anion-exchange columns, combine hydrophobic interactions with electrostatic effects, thereby providing enhanced selectivity for acylated anthocyanin isomers. Under optimized acidic mobile-phase conditions, these systems can improve the separation of structurally similar compounds, including cis- and trans-*p*-coumaroylated petunidin derivatives [26,93].

High-resolution separation is especially useful for chemical characterization and quality standardization. It allows more accurate identification of major and minor anthocyanins, supports the discovery of previously unreported compounds, and provides a basis for extract fingerprints [26]. For commercial products, standardized profiles may improve batch consistency, although specialized chromatographic purification is better suited to analytical, preparative, or high-value extract standardization than to routine food processing. In practical quality-control workflows, high-resolution chromatography serves primarily for structural characterization and fingerprint establishment, while simpler quantitative assays are more feasible for routine batch release. Combining total anthocyanin measurements, color parameters, and characteristic marker peaks offers a pragmatic compromise between analytical accuracy and industrial operability.

### 5.2. Fermentation: Biotransformation and Functional Product Development

Fermentation offers a processing route that changes *L. ruthenicum* rather than merely preserving it. Microorganisms transform sugars, organic acids, phenolics, and other constituents, thereby reshaping flavor, acidity, antioxidant capacity, and bioactive metabolite composition. Compared with raw juice or dried fruit, fermented products may show altered phytochemical profiles and improved bioaccessibility, but the outcome depends strongly on microbial species, substrate composition, fermentation conditions, and post-fermentation treatment.

#### 5.2.1. Vinegar Fermentation

Processing *L. ruthenicum* berries into vinegar represents a value-added strategy that may reduce postharvest losses and expand product diversity. During vinegar fermentation, yeasts and acetic acid bacteria participate in the conversion of sugars into ethanol and subsequently into acetic acid and other organic acids. These metabolic changes alter the physicochemical properties, flavor profile, and functional composition of the final product.

Kinetic studies have shown that fermentation can lead to dynamic changes in anthocyanins, total phenolics, total flavonoids, organic acids, and antioxidant capacity [94]. Although total anthocyanin content may decrease during fermentation because of microbial degradation, pH changes, or oxidative reactions, total phenolic and flavonoid contents may remain stable or even increase. This may be partly attributed to the release of bound phenolics from plant cell-wall structures or the transformation of complex phenolic compounds into more detectable forms.

Beyond this, fermentation may promote the accumulation of functional metabolites, such as gamma-aminobutyric acid and lactic acid, depending on the microbial community and fermentation conditions [94]. Correlation analysis has suggested that the antioxidant activity of fermented *L. ruthenicum* vinegar may be associated not only with residual anthocyanins but also with organic acids, flavonoids, and other fermentation-derived metabolites. These findings indicate that vinegar fermentation may generate a product with a chemical and sensory profile distinct from that of raw juice. Spontaneous fermentation can generate complex flavor profiles yet frequently introduces batch-to-batch inconsistency. Inoculated fermentation improves reproducibility but requires careful strain adaptation to polyphenol-rich substrates. Moreover, vinegar quality should not be judged merely by anthocyanin retention; multiple indicators, including total acid, organic-acid composition, and sensory attributes, should be considered comprehensively. However, further research is needed to optimize fermentation parameters, improve anthocyanin retention, control microbial safety, and evaluate consumer acceptance.

#### 5.2.2. Probiotic Fermentation and Anthocyanin Biotransformation

The principles of fermentation can also be applied to the development of *L. ruthenicum*-based probiotic beverages. The fruit contains fermentable sugars and abundant polyphenols, making it a potential substrate for lactic acid bacteria and other beneficial microorganisms. However, the antimicrobial activity of polyphenols may inhibit certain strains, and therefore appropriate strain selection is essential for successful fermentation.

Lactic acid bacteria and bifidobacteria with suitable acid tolerance, polyphenol tolerance, and beta-glucosidase activity may be particularly useful for *L. ruthenicum* fermentation. Beta-glucosidase-producing strains can hydrolyze glycosidic bonds in anthocyanins and other phenolic glycosides, potentially generating smaller phenolic metabolites such as protocatechuic acid and other anthocyanin-derived degradation products [95,96]. These metabolites may differ from the parent anthocyanins in stability, absorption, and biological activity.

A practical fermentation system must balance several variables at once: anthocyanin preservation, metabolite formation, acid production, sensory quality, and probiotic viability. Proper control of temperature, pH, inoculum size, fermentation time, and substrate concentration may improve flavor, reduce astringency, and alter the bioaccessibility of phenolic compounds [95,96]. Claims of improved bioavailability, however, require evidence from digestion models, uptake assays, animal studies, or human trials.

### 5.3. Stability Enhancement and Encapsulation

The inherent instability of petunidin-derived anthocyanins, especially their sensitivity to neutral pH environments, heat, light, oxygen, and enzymatic degradation, limits their direct application in many food systems [97,98]. In response, stabilization strategies such as encapsulation and copigmentation have been developed to improve physical stability, color retention, and functional preservation of anthocyanin-rich extracts. These approaches may broaden the application of *L. ruthenicum* anthocyanins in beverages, powders, nutraceutical formulations, and natural colorant systems.

Encapsulation techniques, including spray drying, freeze-drying, complex coacervation, liposomes, phytosomes, and protein- or polysaccharide-based nanoparticles, can embed anthocyanin-rich extracts into protective matrices [18,99]. Common wall materials such as maltodextrin, gum arabic, pectin, and proteins may reduce anthocyanin exposure to oxygen, moisture, and light. More advanced protein-polysaccharide coacervates can form dense protective layers around anthocyanin cores through electrostatic interactions, potentially improving storage stability and controlled release. However, claims of targeted intestinal delivery and improved oral bioavailability should be verified using simulated digestion models, release studies, and in vivo pharmacokinetic evidence.

Several stabilization approaches have been investigated for anthocyanins in general and are relevant to *L. ruthenicum* products. Microencapsulation can protect anthocyanins from environmental degradation and allow controlled release in food matrices [18,99]. Copigmentation with phenolic acids, flavonoids, or other food-grade molecules may enhance color intensity and stability through non-covalent interactions [100]. Protein-, polysaccharide-, and lipid-based carriers also offer possible delivery platforms for beverages, powders, and supplements. The main challenge is to choose systems that preserve bioactivity without compromising taste, appearance, regulatory acceptability, or production cost. Copigmentation performance is strongly dependent on pH, molar ratio, temperature, and matrix composition, so it cannot act as a universal stabilization solution for all food systems.

### 5.4. Intelligent Food Packaging Systems

One emerging application of *L. ruthenicum* anthocyanins is their use in active and intelligent food packaging systems. Because anthocyanins exhibit pH-sensitive color changes, they can serve as natural freshness indicators in packaging materials [101]. *L. ruthenicum* anthocyanins typically appear red under acidic conditions, purple or blue under neutral conditions, and green to yellow under alkaline conditions. This color response can be used to monitor pH changes associated with food spoilage, such as acidification during milk deterioration or the accumulation of volatile alkaline compounds during meat and aquatic product spoilage.

To improve practical applicability, *L. ruthenicum* anthocyanins have been incorporated into biopolymer-based composite films [17,102,103]. For example, sodium alginate and konjac glucomannan have been used as film-forming matrices, while tea polyphenols have been introduced to enhance functional performance [17]. The incorporation of *L. ruthenicum* anthocyanins enabled visible color changes in response to food freshness, whereas tea polyphenols improved the mechanical properties and barrier performance of the composite film, including tensile strength, elongation at break, and water vapor permeability. In milk preservation tests, the composite film showed a visible color response associated with pH changes during spoilage, indicating its potential as a freshness-monitoring material [17].

Anthocyanin- and polyphenol-containing films may also contribute to microbial control. Tea polyphenols and *L. ruthenicum* anthocyanins can interact with bacterial surface components and may interfere with cell-wall or membrane integrity [17]. Molecular docking and atomic force microscopy observations support this interpretation, while application tests showed better preservation performance in milk covered with the composite film than in control samples [17]. Long-term stability, migration safety, sensory influence, scalability, and regulatory acceptance remain important requirements for real packaging use. In particular, color-response signals need to be calibrated against specific spoilage markers to avoid misleading interpretation by end-users. Food-contact migration of anthocyanins and auxiliary additives must be quantified, and manufacturing compatibility with film-forming processes should be validated before practical deployment.

### 5.5. Product Diversification and Technical Challenges

The product forms of *L. ruthenicum* have expanded from traditional dried berries and simple infusions to a broader range of value-added products, including juices, fermented beverages, vinegar, solid-state supplements, natural colorants, cosmetic ingredients, and functional food formulations [16]. This diversification reflects the growing interest in *L. ruthenicum* as a source of anthocyanins, polyphenols, polysaccharides, fatty acids, and other bioactive compounds.

Even so, each product category faces specific technical challenges. In beverages, the main problems include anthocyanin degradation, color fading, precipitation, turbidity, and flavor instability. In solid-state supplements, the high sugar content and hygroscopicity of fruit powders may reduce flowability and storage stability. In cosmetic formulations, the major issues include anthocyanin stability, compatibility with other ingredients, and protection against oxidation. As natural colorants, *L. ruthenicum* anthocyanins are attractive because of their intense color and plant origin, but their application is limited by pH sensitivity, light instability, and thermal degradation, especially in neutral or alkaline food systems.

In addition to direct food and cosmetic applications, *L. ruthenicum* extracts may also be used as functional additives in fermentation systems. For example, crude anthocyanin extracts from *L. ruthenicum* have been reported to increase mycelial biomass and exopolysaccharide production during submerged fermentation of edible fungi [104]. This finding suggests a possible role for *L. ruthenicum* extracts in improving fermentation performance or developing novel fermented products. Nevertheless, the underlying mechanisms, optimal dosage, sensory effects, and economic feasibility require further investigation.

Safety and quality control become more important as products move from traditional consumption to concentrated extracts and formulated foods. Solvent residues, microbial contamination, pesticide residues, and toxic elements such as Pb, Cd, As, and Hg should be monitored, particularly because drying and concentration may enrich contaminants along with bioactive compounds. Standardized processing, quality specifications, contaminant limits, and stability testing are therefore essential for commercialization.

In addition, regulatory requirements vary substantially across different product categories. Concentrated extracts demand stricter safety assessment than traditional whole-fruit products, and functional claims require adequate supporting evidence rather than only in-vitro or animal-derived data. Laboratory-scale performance cannot guarantee industrial success; pilot-scale validation covering cost, throughput, and sensory performance is indispensable for practical translation. The major product categories, representative applications, and associated technical challenges of *L. ruthenicum* are summarized in Table 7.

**Table 7:** Product categories, representative products, and technical challenges of *Lycium ruthenicum*.

Product Category	Representative Products	Key Challenges	References
Functional beverages	Juices, fermented beverages, vinegars	Anthocyanin degradation, sedimentation, turbidity, color fading, flavor instability	[94]
Solid-state supplements	Effervescent tablets, capsules, lyophilized powders	Hygroscopicity, poor flowability, caking, high sugar content, storage instability	[10]
Cosmetics	Seed-oil-based creams, anthocyanin-containing formulations	Formulation stability, oxidation, color stability, skin compatibility	[60,72]
Natural colorants	Food-grade colorants for acidic beverages, bakery products, and dairy products	pH sensitivity, light instability, thermal degradation, limited stability in neutral or alkaline systems	[24]
Fermentation additives	Anthocyanin-rich extracts for edible fungi fermentation	Optimal dosage, microbial compatibility, sensory influence, mechanism clarification	[104]
Intelligent packaging	Anthocyanin-loaded freshness indicator films	Anthocyanin migration, color stability, mechanical strength, food-contact safety	[17]

### 5.6. Summary of Food Processing and Product Development

Processing research on *L. ruthenicum* is moving from simple drying and extraction toward more diversified, product-oriented technologies. Green extraction can improve anthocyanin recovery, fermentation can reshape flavor and metabolite composition, encapsulation can support stability, and intelligent films can use anthocyanin color changes for freshness monitoring. These directions are connected by the same practical challenge: maintaining bioactive compounds in forms that are stable, safe, acceptable, and scalable.

Despite these advances, several challenges remain. Anthocyanin degradation, pH sensitivity, oxidation, poor storage stability, inconsistent raw material quality, and insufficient safety evaluation continue to limit large-scale industrial application. Future development should focus on standardized raw material control, food-grade extraction and stabilization technologies, encapsulation systems, fermentation optimization, safety assessment, and product-specific quality standards. Addressing these issues will support the rational development of *L. ruthenicum* as a high-value functional food resource, natural colorant, and food-packaging material. Future progress should shift from proof-of-concept laboratory investigations toward reproducible, scalable product systems. An integrated framework covering raw-material control, food-grade processing, chemical characterization, safety evaluation, and regulatory compliance is required to fully realize the industrial potential of *L. ruthenicum*.

## 6. Genomic Resources and Their Relevance to Quality Formation and Standardization

The stress tolerance and phytochemical richness of *L. ruthenicum* are linked to its genetic background and regulatory networks. As a halophytic and xerophytic shrub adapted to saline-alkaline, arid, and high-ultraviolet environments, it provides a useful system for studying plant adaptation and secondary metabolite regulation. Chloroplast genome sequencing, transcriptomic profiling, and stress-response analyses now offer molecular resources for understanding evolution, fruit development, anthocyanin biosynthesis, and stress tolerance.

Genomic information provides an important link between the genetic basis of *L. ruthenicum* and its development as a standardized functional food resource. Genome studies can facilitate species authentication and provide valuable genetic resources for *L. ruthenicum* characterization [105]. Transcriptomic and metabolomic analyses have further revealed the molecular basis of anthocyanin accumulation and other quality-related traits during fruit development [106]. Therefore, genomic research is not only important for understanding evolutionary relationships and stress adaptation but also provides a foundation for future quality-oriented breeding and the development of more consistent *L. ruthenicum*-derived food ingredients.

### 6.1. Chloroplast Genome and Evolutionary Insights

The chloroplast genome is an important resource for plant phylogenetic analysis, species identification, and evolutionary studies. The complete chloroplast genome of *L. ruthenicum* has been sequenced and characterized, showing the typical quadripartite structure found in most angiosperms. It consists of a large single-copy region, a small single-copy region, and two inverted repeat regions.

The chloroplast genome of *L. ruthenicum* is a circular molecule of approximately 155–156 kb. It contains genes related to photosynthesis, transcription, translation, and chloroplast maintenance, including *protein-coding* genes, *rRNA* genes, and *tRNA* genes [105,107]. The overall GC content has been reported to be approximately 37.9%, indicating an AT-rich nucleotide composition [105,107]. Such nucleotide composition may influence codon usage patterns and genome evolution; however, whether it directly contributes to stress adaptation requires further experimental validation.

Phylogenetic analyses based on chloroplast genome data have clarified the taxonomic position of *L. ruthenicum* within the family Solanaceae. *L. ruthenicum* clusters closely with related *Lycium* species, including *L. barbarum* and *L. chinense*, supporting their close evolutionary relationship [107,108]. At the same time, sequence variations, such as single nucleotide polymorphisms and insertion-deletion sites in intergenic spacer regions, provide useful molecular markers for distinguishing *L. ruthenicum* from related species. These chloroplast genome resources may therefore be valuable for germplasm identification, phylogenetic reconstruction, and the development of molecular markers for breeding and quality control.

To further illustrate the evolutionary position of *L. ruthenicum* within the genus *Lycium* and provide a genomic framework for species comparison, a phylogenetic tree based on chloroplast genome sequences was constructed (Figure 8). In this analysis, 136 single-copy genes from representative species were used, including eight Solanaceae species, namely *Nicotiana tabacum*, *Solanum tuberosum*, *Capsicum annuum*, *Solanum melongena*, *Petunia × hybrida*, *Solanum lycopersicum*, *L. ruthenicum*, and *L. barbarum*, together with *Arabidopsis thaliana* and *Ginkgo biloba* as reference species and the outgroup, respectively. As shown in the phylogenetic tree, after the divergence of G. *biloba*, the sampled angiosperms were separated into two major lineages. A. *thaliana* was placed outside the asterid-dominated lineage, whereas Petunia and other Solanaceae species were grouped within the asterid/Solanaceae branch. Within Solanaceae, *L. barbarum* and *L. ruthenicum* formed a monophyletic *Lycium* clade, supporting their close evolutionary relationship. This *Lycium* clade was positioned close to the Solanum lineage represented by tomato, eggplant, potato, and related species. The relatively short branch length between *L. barbarum* and *L. ruthenicum* further suggests their close phylogenetic affinity. These results are consistent with previous chloroplast genome-based analyses and provide additional molecular evidence supporting the phylogenetic placement of *L. ruthenicum* within the genus *Lycium*.

Some studies connect *Lycium* diversification with environmental changes in arid and semi-arid regions, including geological and climatic shifts related to the Qinghai–Tibet Plateau and northwest China [109]. This interpretation is plausible but should be treated carefully. A stronger explanation will require nuclear genomic data, population genetics, ecological evidence, and functional analyses of stress-tolerance traits.

### 6.2. Transcriptomic Regulation of Fruit Development

Beyond species identification and evolutionary characterization, genomic and transcriptomic studies have provided insights into the molecular mechanisms underlying important quality-related traits of *L. ruthenicum*, particularly the accumulation of anthocyanins responsible for its characteristic pigmentation and functional value. Among these traits, fruit development represents a key biological process determining phytochemical composition and fruit quality. Fruit development in *L. ruthenicum* involves coordinated changes in morphology, color formation, primary metabolism, and secondary metabolite accumulation. Transcriptomic studies across green, color-turning, and fully ripe stages provide a way to follow these changes at the gene-expression level, especially during anthocyanin accumulation.

#### 6.2.1. WRKY Transcription Factor Family

WRKY transcription factors are important regulators involved in plant development, stress responses, and secondary metabolism. Transcriptome-wide analysis has identified 73 potential WRKY members in *L. ruthenicum*, among which 44 proteins containing the conserved WRKY domain were further confirmed [22]. Phylogenetic analysis classified these LrWRKY proteins into three major groups and several subgroups, with Group II representing the largest group.

Expression analysis across three fruit developmental stages, including the green stage, color-turning stage, and fully ripe stage, showed that *LrWRKY* genes exhibit stage-specific expression patterns [22]. Among the confirmed *LrWRKY* genes, 23 showed relatively high expression levels in at least one developmental stage. For example, *LrWRKY20* maintained relatively high expression throughout fruit development, whereas *LrWRKY8* and *LrWRKY27* showed decreasing expression from the early to late stage. RT-qPCR validation further indicated that several genes, including *LrWRKY20*, *LrWRKY21*, *LrWRKY28*, *LrWRKY30*, and *LrWRKY31*, displayed increasing expression patterns during fruit maturation [22].

These expression patterns suggest that specific *LrWRKY* genes may participate in fruit development, ripening, and secondary metabolite regulation. Genes with high or increasing expression during later developmental stages may be associated with maturation-related processes, including color development and metabolic reprogramming. Homology also provides useful clues: some Group I *LrWRKYs* resemble *WRKY* proteins involved in developmental regulation, while Group IIc members are related to *WRKYs* implicated in fruit development and coloration in other species [22]. Functional validation through overexpression, silencing, or genome editing is still needed. From a functional-food perspective, elucidating these developmental regulators may help identify germplasm with more stable fruit pigmentation and phytochemical composition, thereby supporting quality-oriented breeding and raw-material standardization.

#### 6.2.2. Regulation of Anthocyanin Biosynthesis

Following the developmental patterns described above, the most conspicuous change during *L. ruthenicum* fruit ripening is the transition from green to dark purple-black, accompanied by a pronounced increase in anthocyanin accumulation. Transcriptomic analyses of green, color-turning, and fully ripe fruits have identified extensive transcriptional reprogramming in the phenylpropanoid and flavonoid pathways [19]. More recent integrated metabolomic and transcriptomic profiling across five developmental stages further showed that anthocyanin accumulation accelerates after the onset of coloration and reaches its highest level in mature fruits, with delphinidin- and petunidin-related metabolites contributing substantially to the developmental pigment profile [106].

At the biochemical level, anthocyanin biosynthesis begins with the conversion of phenylalanine through the phenylpropanoid pathway, followed by a series of reactions within the flavonoid pathway. Candidate structural genes identified in *L. ruthenicum* include *PAL*, *C4H*, *4CL*, *CHS*, *CHI*, *F3H*, *F3′H*, *F3′5′H*, *DFR*, *ANS/LDOX*, and *UFGT* (Figure 9) [19,106]. Among these, *F3′5′H*, *DFR*, *ANS*, and *UFGT* show particularly close associations with anthocyanin accumulation during fruit maturation [106]. Collectively, these expression patterns are consistent with preferential accumulation through the delphinidin–petunidin branch, in agreement with the petunidin-rich anthocyanin profile of mature black fruits [5]. Nevertheless, most structural genes have been identified through differential-expression and correlation analyses, and their individual biochemical functions in *L. ruthenicum* have not all been experimentally verified.

Substantial progress has been made in defining the transcriptional hierarchy controlling this pathway. Comparative analyses of black- and white-fruited *L. ruthenicum* demonstrated that *LrAN2-like*, *LrAN1b*, and *LrAN11* interact to form a MYB–bHLH–WD40 complex involved in the regulation of anthocyanin biosynthesis. *LrAN2-like* was also shown to regulate the promoters of the structural genes *LrDFR* and *LrANS* [20]. Subsequent functional analyses revealed a hierarchical regulatory system in which the *LrAN2-like*–*LrJAF13*–*LrAN11* complex activates *LrAN1b* expression, whereas the *LrAN2-like*–*LrAN1b*–*LrAN11* complex regulates downstream anthocyanin-biosynthetic genes [110]. A 229-bp deletion in the *LrAN1b* promoter of white-fruited material removes key MYB- and bHLH-binding elements and markedly reduces *LrAN1b* expression. The regulatory importance of *LrAN1b* is further supported by transient complementation, which partially restores pigmentation in white fruits, and by virus-induced gene silencing, which decreases anthocyanin accumulation and the expression of structural genes in black fruits [110]. These findings provide direct evidence that *LrAN1b* is a central positive regulator of black-fruit pigmentation.

Beyond the formation of the anthocyanidin backbone, the structural diversity of *L. ruthenicum* anthocyanins is generated through glycosylation, methylation, and acylation. Comparative transcriptomic analyses have identified several candidate modification-related genes that are more highly expressed in black than in white fruits, including *LrUFGT*, *Lr3GT*, *LrUGT75C1*, *LrAOMT*, and *Lr3AT* [20]. *LrUFGT* and *Lr3GT* are associated primarily with 3-*O*-glycosylation, whereas *LrUGT75C1* has been proposed to contribute to additional glycosylation events, potentially including modification at the 5-*O* position. *LrAOMT* is a candidate anthocyanidin *O*-methyltransferase associated with the formation of petunidin- and malvidin-related products, while *Lr3AT* is a putative acyltransferase that may contribute to the extensive acylation characteristic of black-goji anthocyanins [5,20]. Because the precise sequence of these reactions has not been fully resolved, glycosylation, methylation, and acylation are more appropriately represented as an interconnected post-biosynthetic modification network rather than as a single invariant linear pathway.

Following synthesis and modification, anthocyanins must be transported into the vacuole for stable accumulation. *GST*-, *MRP/ABC*-, and *MATE*-related genes have been identified as candidate components of this transport system on the basis of sequence annotation and co-expression with anthocyanin-related genes [20]. Their specific substrate preferences and transport functions in *L. ruthenicum*, however, remain to be established experimentally. A further regulatory layer may be provided by WRKY transcription factors. Several *LrWRKY* genes exhibit stage-dependent expression during fruit development [22], and integrated developmental analyses have identified WRKY candidates whose expression correlates with anthocyanin accumulation [109]. Although these findings suggest a possible role in pigment regulation, direct binding of LrWRKY proteins to anthocyanin-biosynthetic gene promoters has not yet been demonstrated. WRKY involvement should therefore be regarded as putative, in contrast to the more firmly established role of the hierarchical MYB–bHLH–WD40 network.

A well-defined developmental regulatory network governs anthocyanin biosynthesis in *L. ruthenicum* fruits. By comparison, direct molecular evidence linking environmental stressors—such as salinity, drought, or high-ultraviolet exposure—to specific anthocyanin regulators remains limited. These experimentally supported and proposed relationships are summarized in Figure 9.

### 6.3. Molecular Mechanisms of Stress Tolerance

*L. ruthenicum* is well adapted to saline-alkaline, drought-prone, and high-radiation environments, making it a useful species for studying plant tolerance to abiotic stress. Its stress tolerance is likely supported by multiple coordinated mechanisms, including ion homeostasis, osmotic adjustment, antioxidant defense, stress-responsive transcriptional regulation, and secondary metabolite accumulation [21,36,37,111].

Salinity-responsive transcriptomic and metabolomic studies indicate coordinated changes in hormone signaling, ion transport, redox homeostasis, osmotic adjustment, and secondary metabolism in *L. ruthenicum* [21,36,37]. Functional characterization identified *LrAMADH1* as the bona fide BADH involved in glycine betaine biosynthesis in *L. ruthenicum*, with its salt-inducible expression suggesting a role in osmotic stress adaptation [111]. Although *NHX*- and *SOS*-related components are plausible contributors to ionic homeostasis, their specific functions in *L. ruthenicum* require direct genetic and biochemical validation.

Osmotic adjustment is another key component of stress tolerance. Genes involved in the biosynthesis of compatible solutes, such as choline monooxygenase and betaine aldehyde dehydrogenase, may be upregulated under stress conditions, contributing to the accumulation of glycine betaine [111]. Glycine betaine can function as an osmoprotectant by stabilizing proteins, membranes, and photosynthetic structures under salinity and drought stress. This mechanism may partly explain the strong environmental adaptability of *L. ruthenicum*.

Transcription factors provide another layer of stress regulation. *WRKY* genes, including *LrWRKY2* and *LrWRKY23*, respond rapidly to drought or salt stress [22]. By binding W-box elements in the promoters of stress-responsive genes, these regulators may influence defense responses, antioxidant enzymes, and cellular protection. The overlap between developmental and stress-response networks helps explain why environmental stress can affect secondary metabolite accumulation, although moderate stress may enhance some phytochemicals while severe stress can reduce growth, yield, and fruit quality.

### 6.4. Summary of Genomic Resources and Stress Biology

Together, genomic and transcriptomic studies have expanded understanding of the evolution, development, and stress adaptation of *L. ruthenicum*. Chloroplast genome analyses support phylogenetic classification and marker development, while transcriptomic studies reveal candidate regulators of ripening and anthocyanin biosynthesis. Stress-response analyses further point to ion homeostasis, osmotic adjustment, antioxidant defense, and transcriptional regulation as major adaptive mechanisms.

From a functional-food perspective, the practical value of these genomic resources lies in supporting species authentication, germplasm characterization, and quality-oriented breeding strategies, which may contribute to the selection of raw materials with more consistent phytochemical profiles. Integration of genomic and transcriptomic information with metabolomic profiling and standardized chemical analysis may further improve traceability and quality consistency of *L. ruthenicum*-derived ingredients, thereby strengthening the foundation for future quality standardization and industrial application [105,106,109].

## 7. Conclusions and Future Perspectives

*Lycium ruthenicum Murr*. is a valuable medicinal and edible plant that links traditional dietary knowledge with modern food science, nutrition, and biotechnology. Its significance lies not only in its high antioxidant capacity but also in its complex phytochemical matrix, including acylated petunidin-derived anthocyanins, polysaccharides, phenolic acids, flavonoids, unsaturated fatty acids, tocopherols, phytosterols, minerals, and nitrogenous compounds. These constituents underpin its nutritional value, distinctive color, reported biological activities, and product-development potential. Studies reviewed here suggest antioxidant, anti-inflammatory, metabolic-regulatory, neuroprotective, hepatoprotective, gut microbiota-modulating, and anti-fatigue effects. However, the current evidence base remains uneven: many findings derive from crude extracts, broad fractions, in vitro assays, or animal models, whereas standardized raw materials, harmonized analytical methods, realistic product matrices, human bioavailability data, safety assessment, clinical validation, regulatory feasibility, and industrial scalability remain insufficiently developed. Therefore, future development of *L. ruthenicum* should move from descriptive characterization and proof-of-concept bioactivity testing toward translational research systems that connect raw material, processing, chemical standardization, biological relevance, product safety, and market feasibility.

Future research should first strengthen standardization across the entire research and product-development chain. Minimum quality specifications should be established for berries and primary processed materials, covering botanical authentication, maturity, moisture, marker compounds, contaminants, and microbial limits. Extraction, quantification, and structural characterization protocols should be harmonized, and confirmed structures should be clearly distinguished from tentative assignments. Anthocyanin and polysaccharide stability should be evaluated in intended product matrices such as beverages, fermented products, powders, dairy systems, and packaging films, with food-grade stabilization strategies—including encapsulation, copigmentation, oxygen control, light-protective packaging, and mild processing—validated under realistic storage and distribution conditions. Research should also move from crude extracts and broad fractions toward reproducible, structurally defined preparations, combining purified-compound studies with standardized matrix-based preparations to clarify structure–activity relationships and matrix effects.

Future work should further integrate bioavailability, safety, clinical validation, regulatory feasibility, and industrial scalability. Simulated digestion, cellular transport, animal pharmacokinetics, and human postprandial studies should be combined to identify the forms and metabolites that reach systemic circulation and target tissues. Safety assessment should cover solvent residues, extraction aids, microbial and pesticide contamination, toxic elements, oxidation products, and migration from packaging materials. Standardized products should then be tested in randomized, double-blind, placebo-controlled trials with clinically meaningful endpoints, including oxidative stress, inflammation, metabolic, microbiota, cognitive, visual, and exercise-fatigue parameters. In parallel, sustainable cultivation and quality-oriented breeding, pilot- and production-scale validation of extraction, stabilization, fermentation, encapsulation, and intelligent packaging, together with techno-economic and regulatory assessment, will determine whether laboratory findings can be translated into functional foods, natural colorants, nutraceutical ingredients, and smart packaging materials. Addressing these priorities will provide a realistic path from the promising chemistry of *L. ruthenicum* to evidence-based applications.

## Figures and Tables

**Figure 1:**
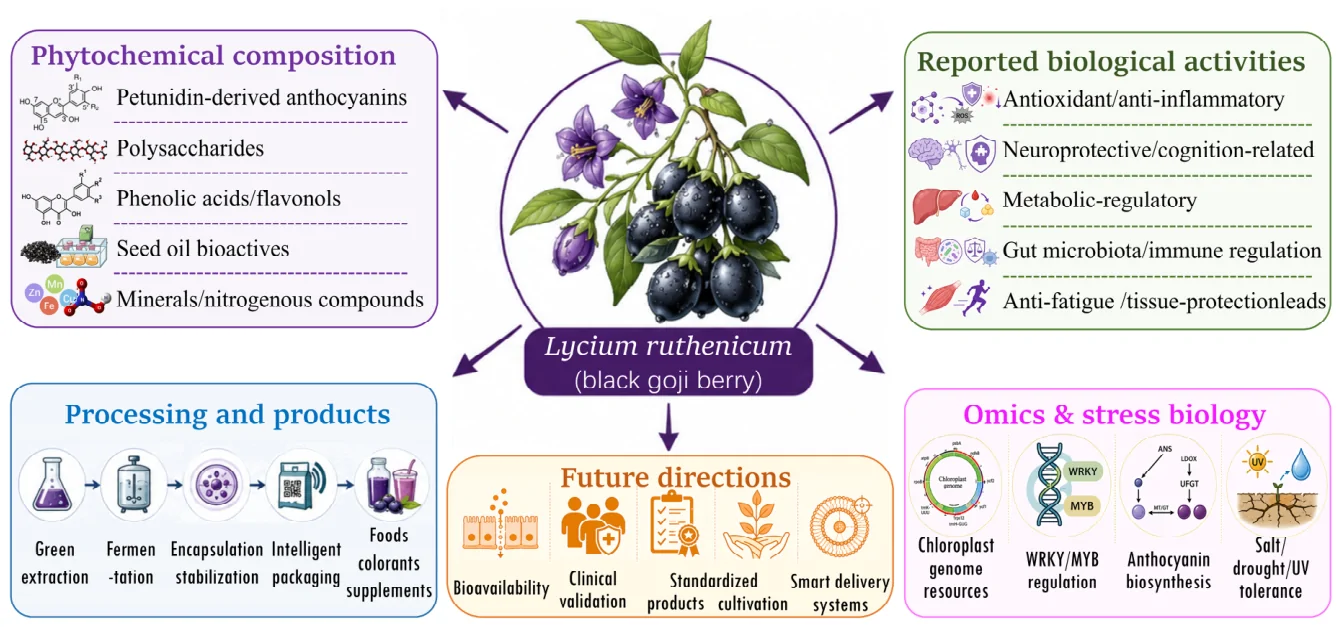
Graphical overview of *Lycium ruthenicum* as a functional food resource.

**Figure 2:**
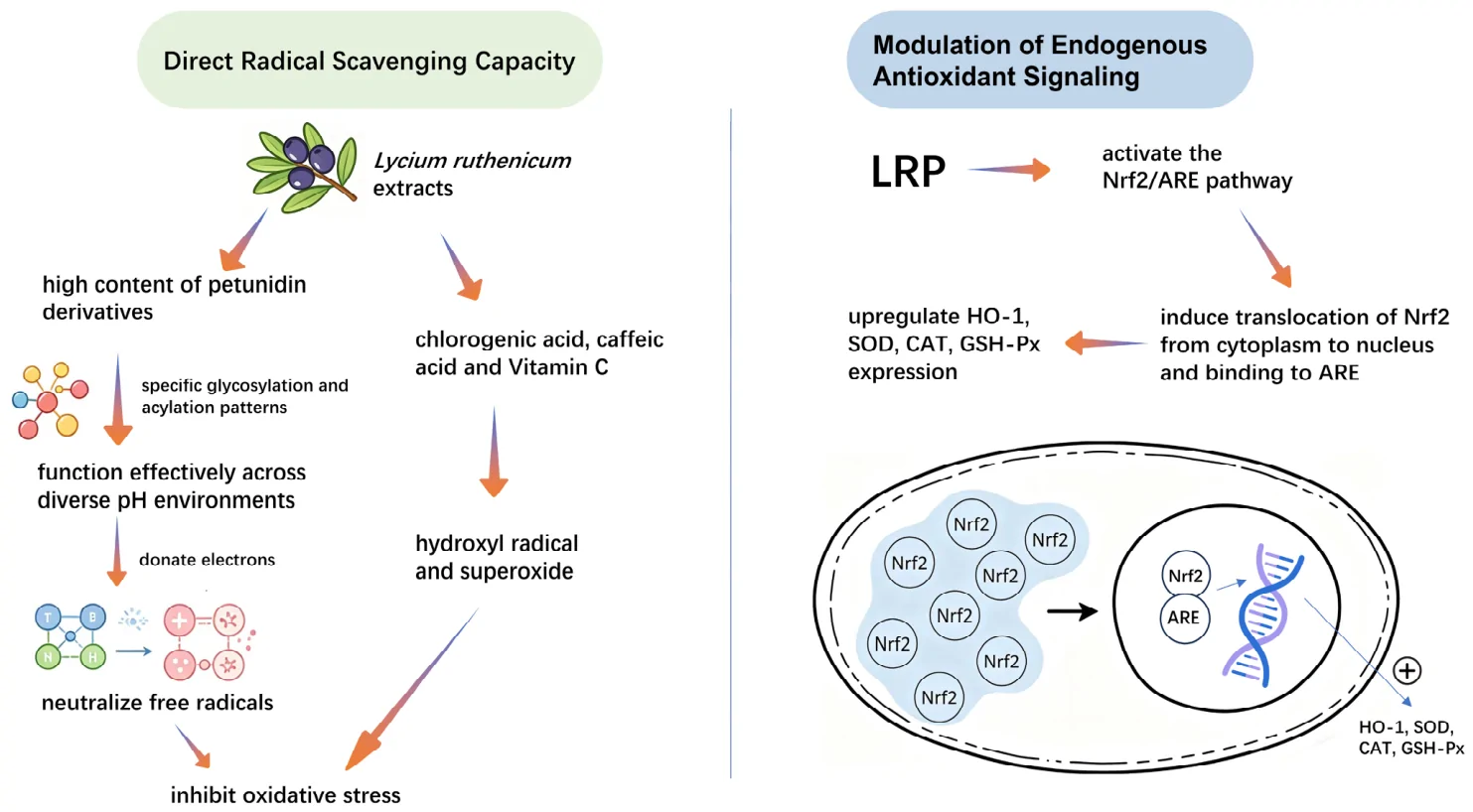
Proposed antioxidant mechanisms of *L. ruthenicum* bioactive constituents. *L. ruthenicum* extracts may exert antioxidant effects through direct radical scavenging and modulation of endogenous antioxidant signaling, particularly Nrf2/ARE-mediated antioxidant defense.

**Figure 3:**
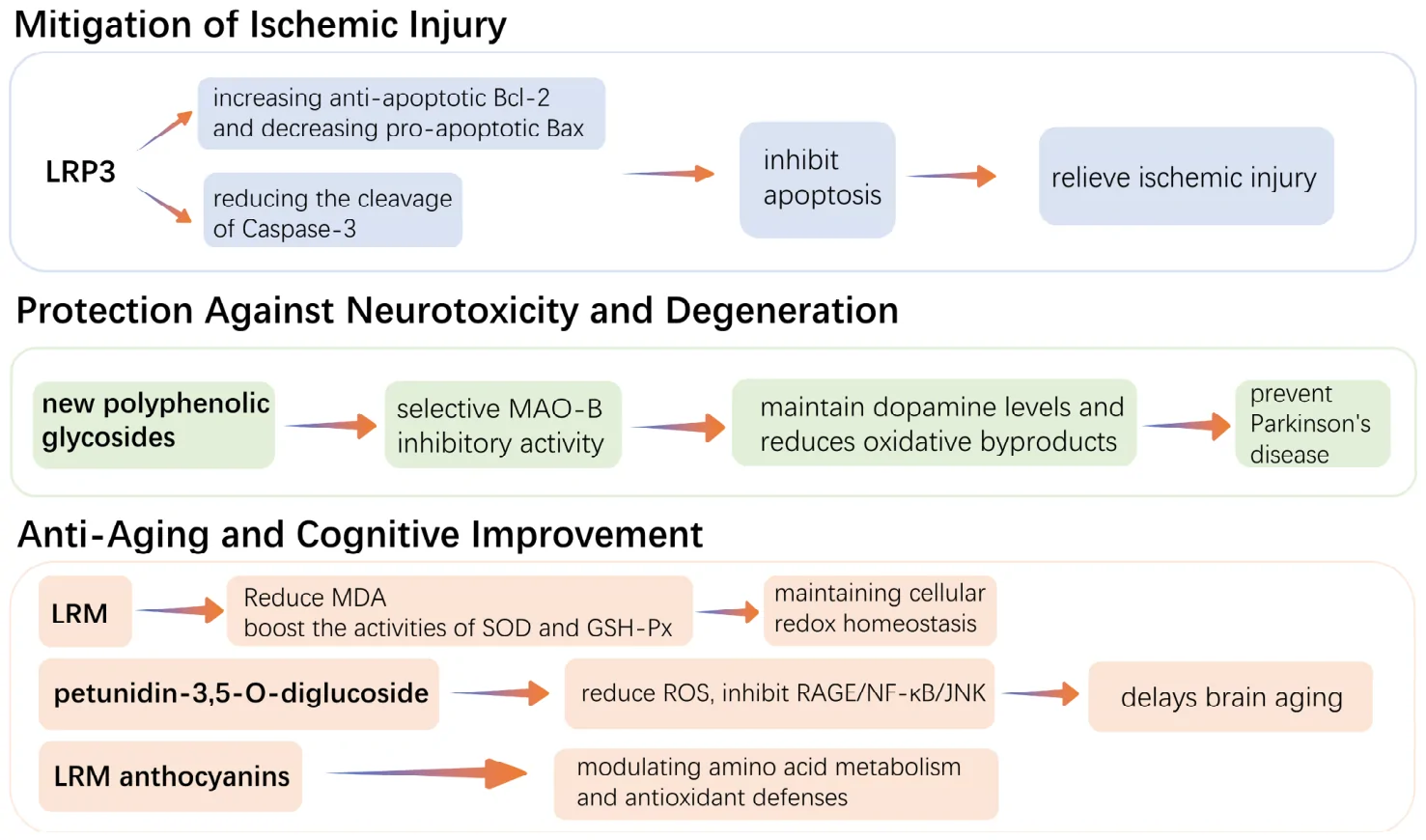
Proposed neuroprotective mechanisms of *L. ruthenicum* bioactive constituents. LRP3 may mitigate ischemia-like neuronal injury by regulating apoptosis-related proteins; polyphenolic glycosides may contribute to dopaminergic neuroprotection; and anthocyanin-rich extracts may improve aging-related cognitive parameters by reducing oxidative damage and inflammation.

**Figure 4:**
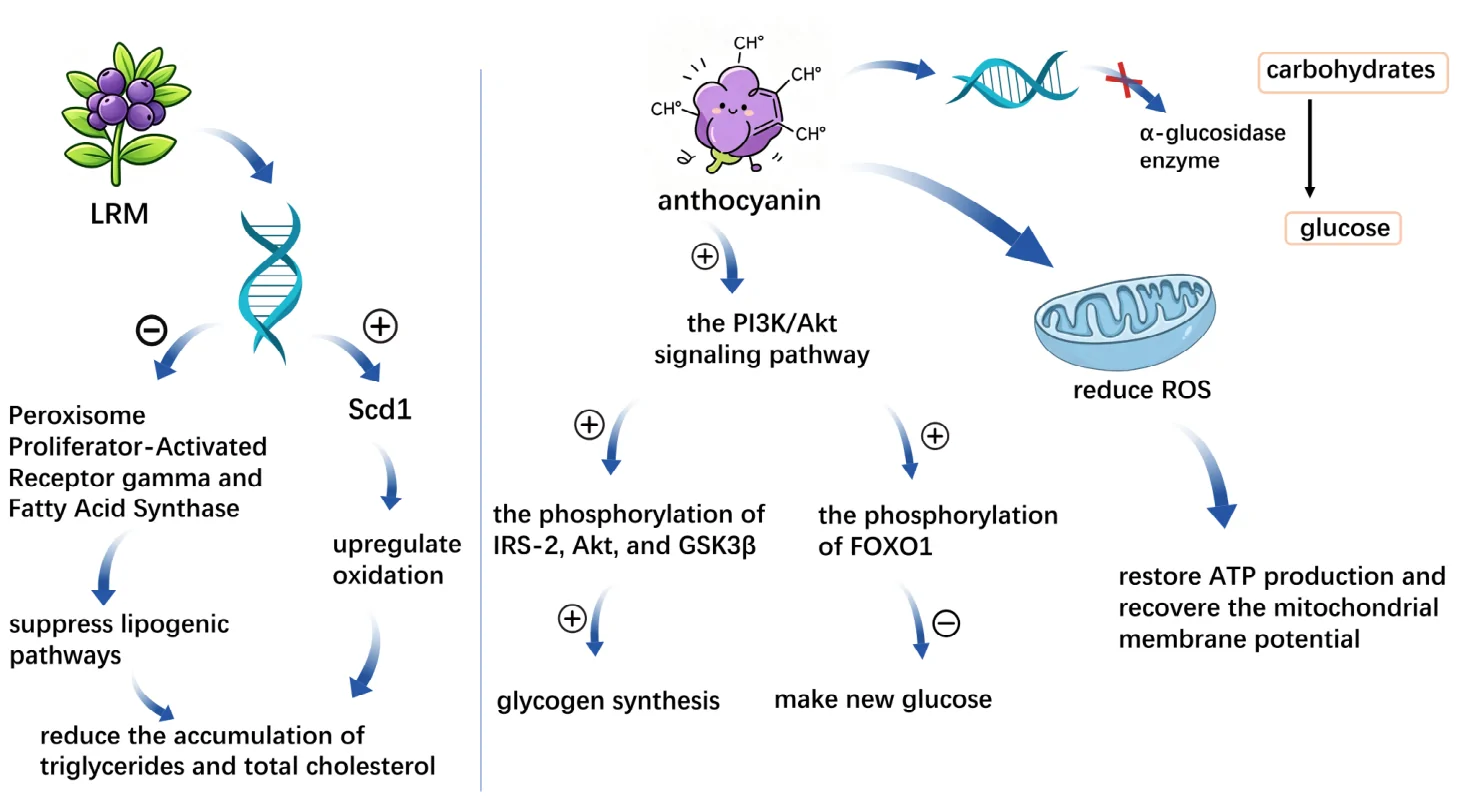
Proposed mechanisms underlying the metabolic-regulatory effects of *L. ruthenicum*. *L. ruthenicum* extracts may regulate hepatic lipid metabolism, while anthocyanins may improve glucose metabolism by inhibiting alpha-glucosidase, activating PI3K/Akt insulin signaling, reducing ROS production, and improving mitochondrial function.

**Figure 5:**
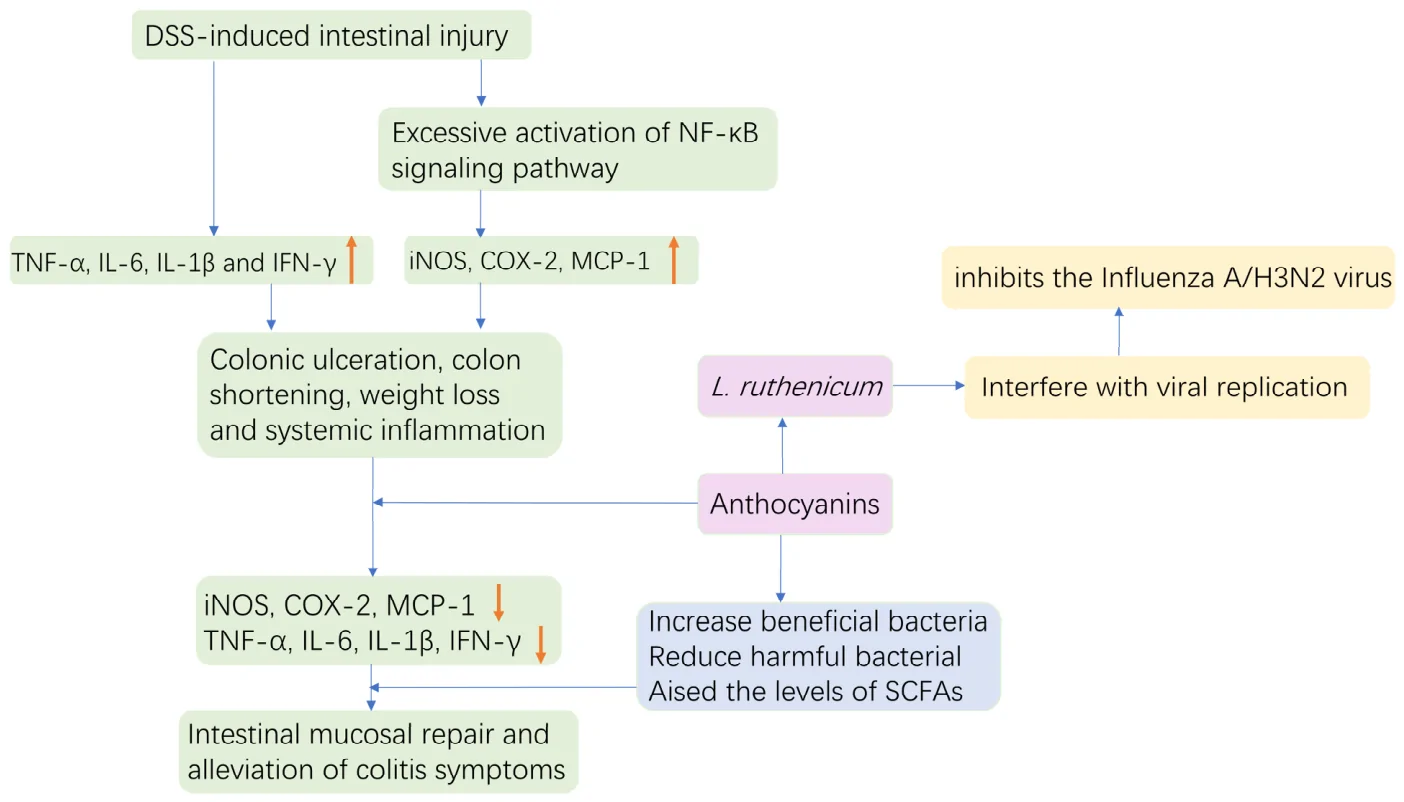
Proposed mechanisms underlying the immunomodulatory and gut health-related effects of *L. ruthenicum*. In DSS-induced colitis models, *L. ruthenicum* anthocyanins may attenuate intestinal inflammation, remodel gut microbiota composition, increase SCFA production, and support intestinal mucosal repair. In vitro antiviral activity against influenza A/H3N2 has also been reported. Orange upward and downward arrows indicate increased and decreased levels, respectively, whereas blue arrows indicate the direction of the corresponding biological processes or regulatory relationships.

**Figure 6:**
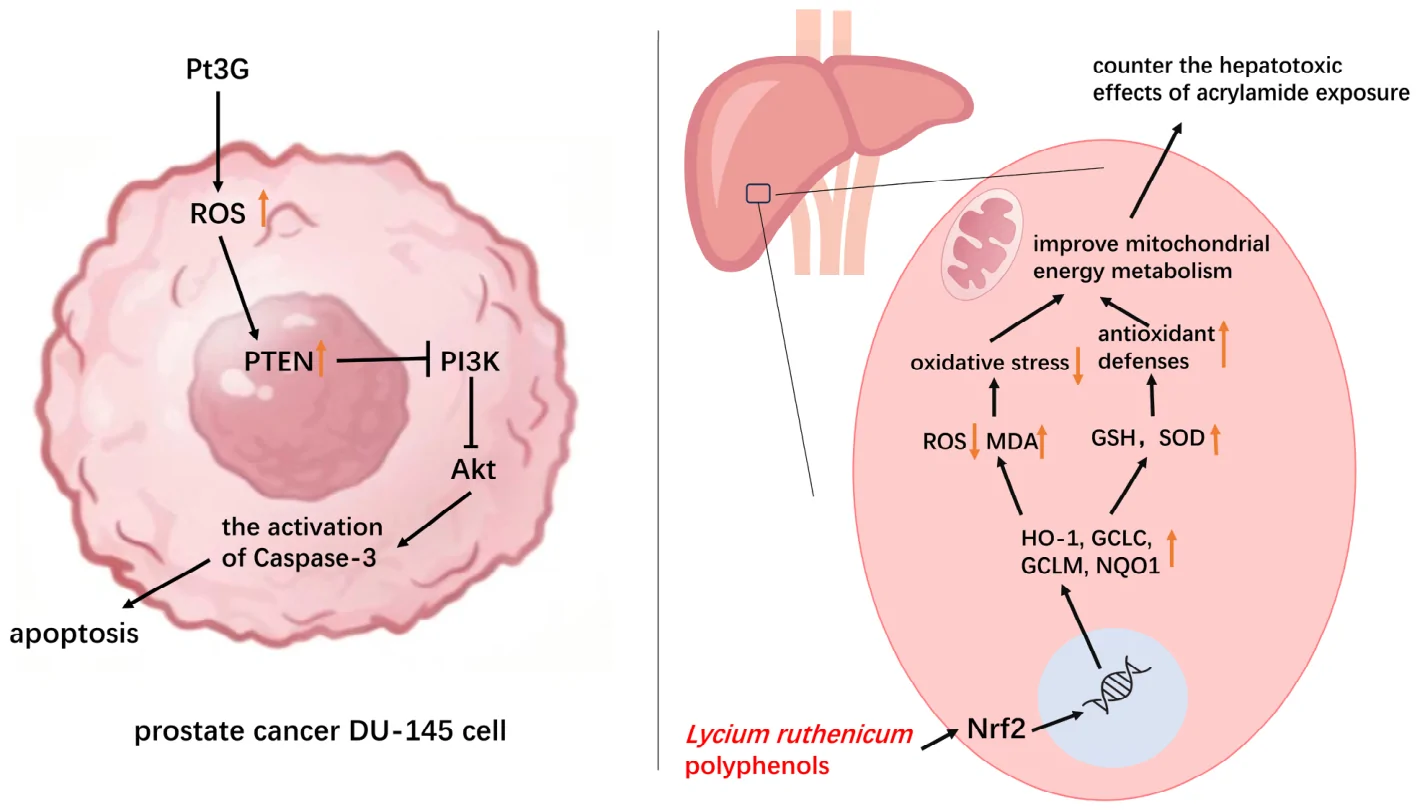
Proposed mechanisms underlying the antiproliferative and hepatoprotective effects of *L. ruthenicum* bioactive constituents. Petunidin-3-*O*-glucoside may induce apoptosis in DU145 prostate cancer cells, whereas *L. ruthenicum* polyphenols may activate Nrf2-related antioxidant signaling and alleviate acrylamide-induced hepatotoxicity. Orange upward and downward arrows indicate increased and decreased levels, respectively; black arrows indicate the direction of the proposed biological effects, T-shaped lines indicate inhibitory effects, and red italic text denotes *L. ruthenicum* polyphenols.

**Figure 7:**
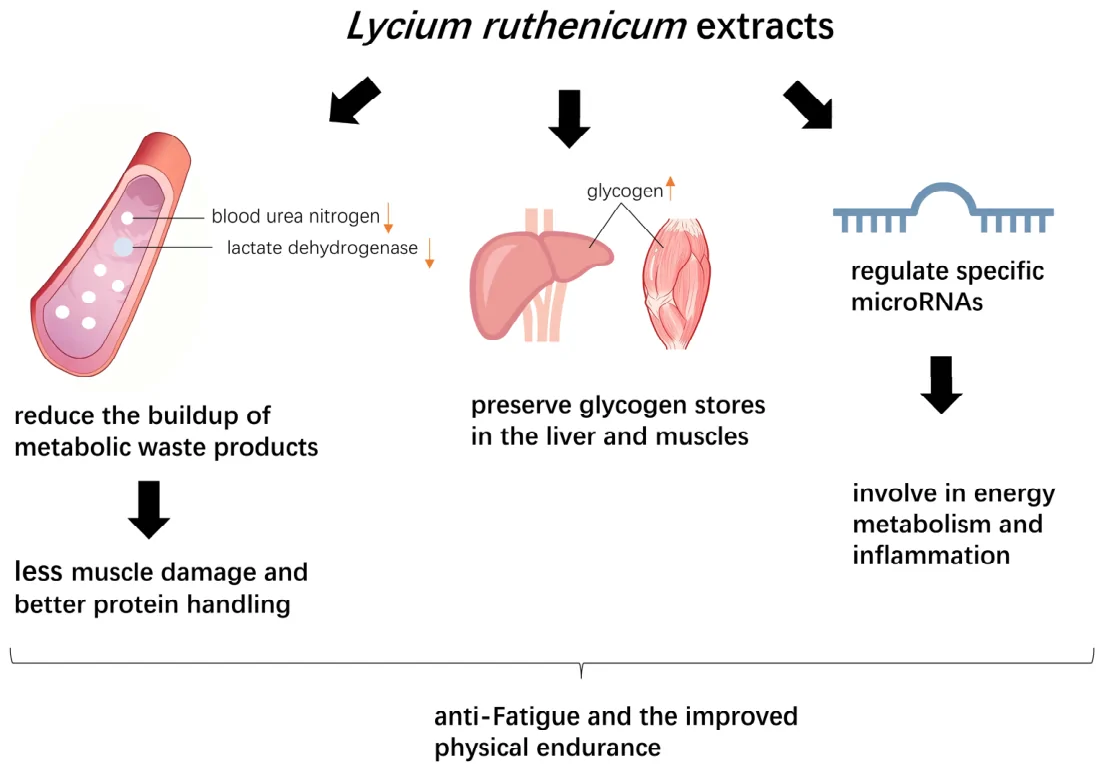
Proposed mechanisms underlying the anti-fatigue and physical performance-related effects of *Lycium ruthenicum* extracts.

**Figure 8:**
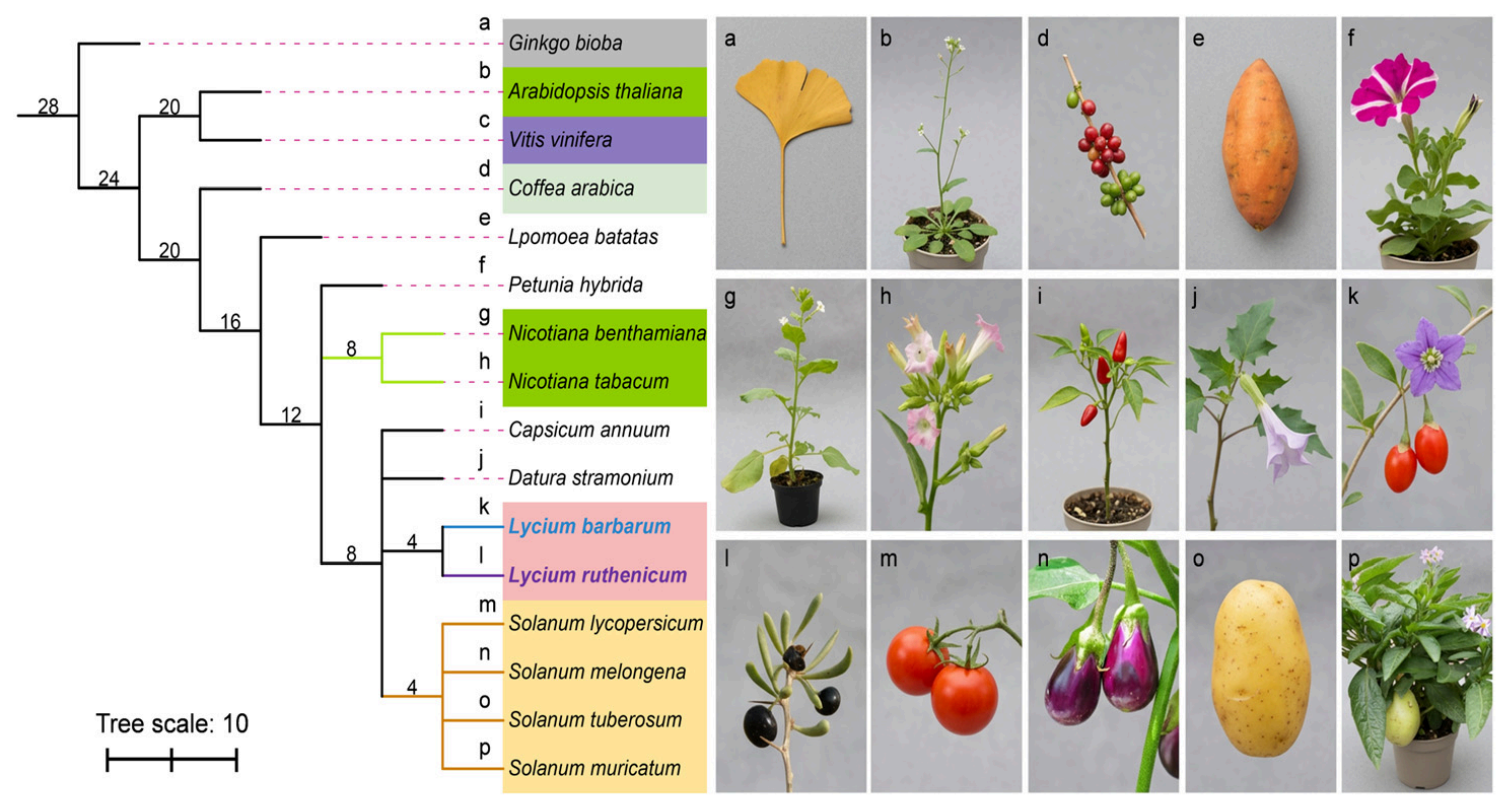
Phylogenetic trees and divergence times of the eight species examined.

**Figure 9:**
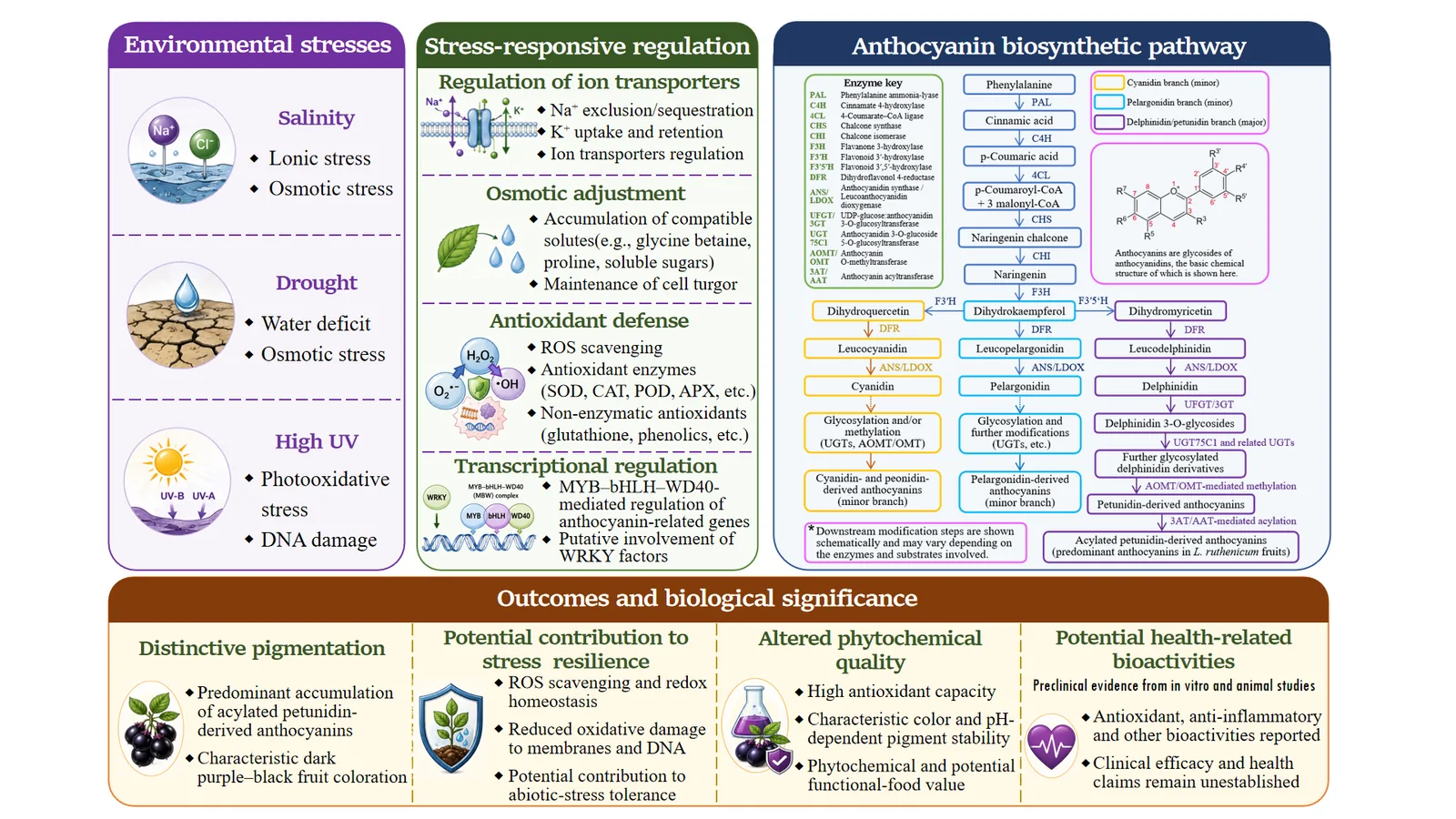
Integrated framework linking abiotic-stress responses, anthocyanin biosynthesis, and phytochemical traits in *Lycium ruthenicum*. The schematic summarizes the proposed relationships among environmental stresses, stress-responsive regulation, and the phenylpropanoid–flavonoid pathway. The delphinidin–petunidin branch and downstream glycosylation, methylation, acylation, and vacuolar accumulation are highlighted as major determinants of the characteristic petunidin-rich pigment profile and dark purple-black coloration of mature fruits. WRKY involvement and the proposed links with drought and ultraviolet exposure remain to be functionally validated, whereas the reported health-related activities are supported predominantly by preclinical evidence.

**Table 1:** Evidence classification framework used in this review.

Evidence Tier	Definition	Examples
Tier 1	Human evidence	Clinical/intervention studies
Tier 2	In vivo evidence	Mouse, rat, other animal models
Tier 3	In vitro biological evidence	Cell, primary cell, tissue models
Tier 4	Chemical/biochemical evidence	DPPH, ABTS, FRAP, enzyme assays

**Table 2:** Major anthocyanins identified in *Lycium ruthenicum* fruits.

No.	Representative Compound	Structural Formula	Anthocyanidin Class	Relative Abundance/Content	Identification Method	References
1	petunidin 3-rutinoside-5-glucoside	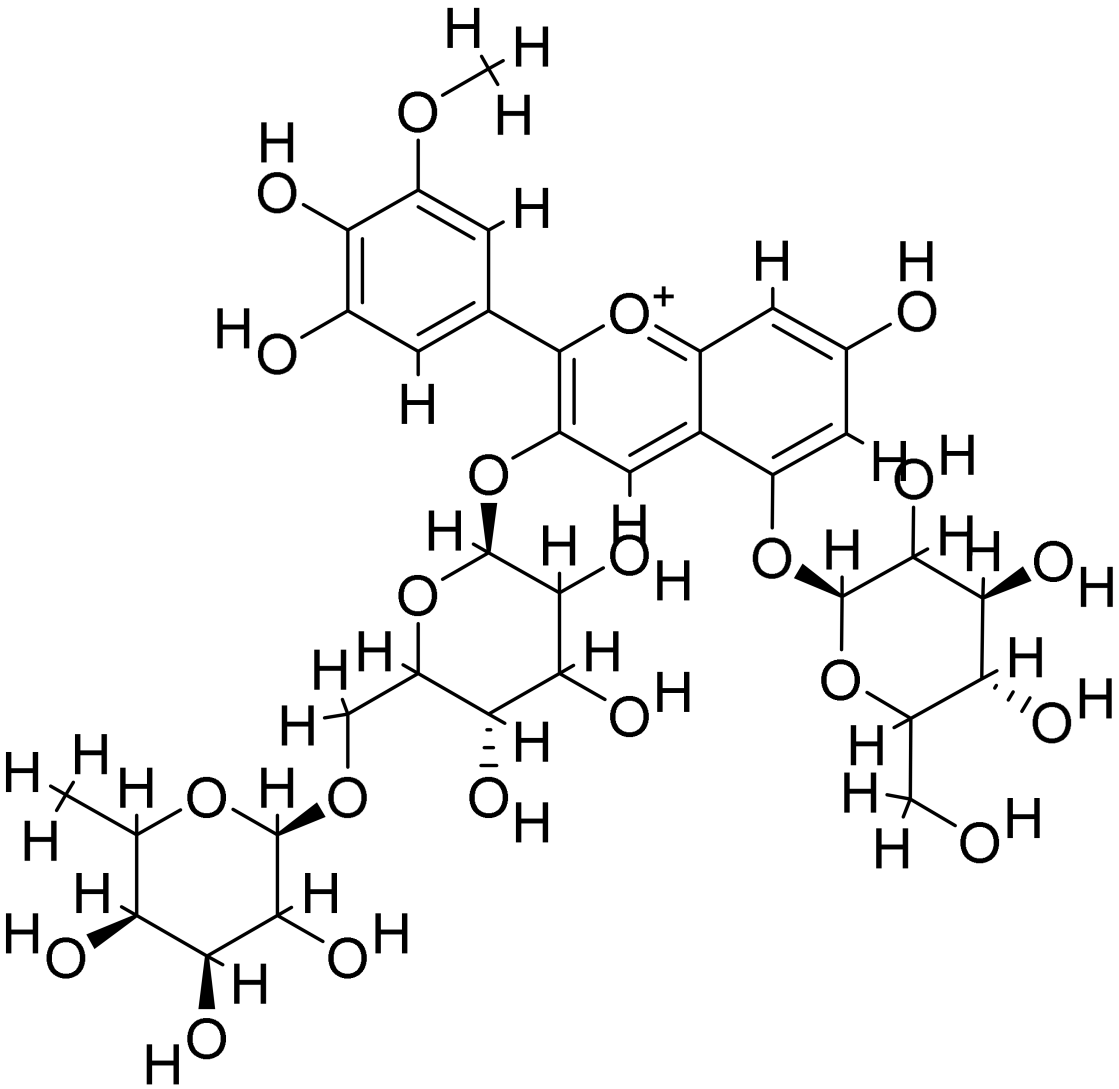	petunidin	82.7%	HPLC-DAD-ESI-MS	[28]
2	petunidin 3-*O*-[6-*O*-(4-*O*-(4-*O*-(β-D-glucopyranosyl)-cis-*p*-coumaroyl)-α-L-rhamnopyranosyl)-β-D-glucopyranoside]-5-*O*-[β-D-glucopyranoside]	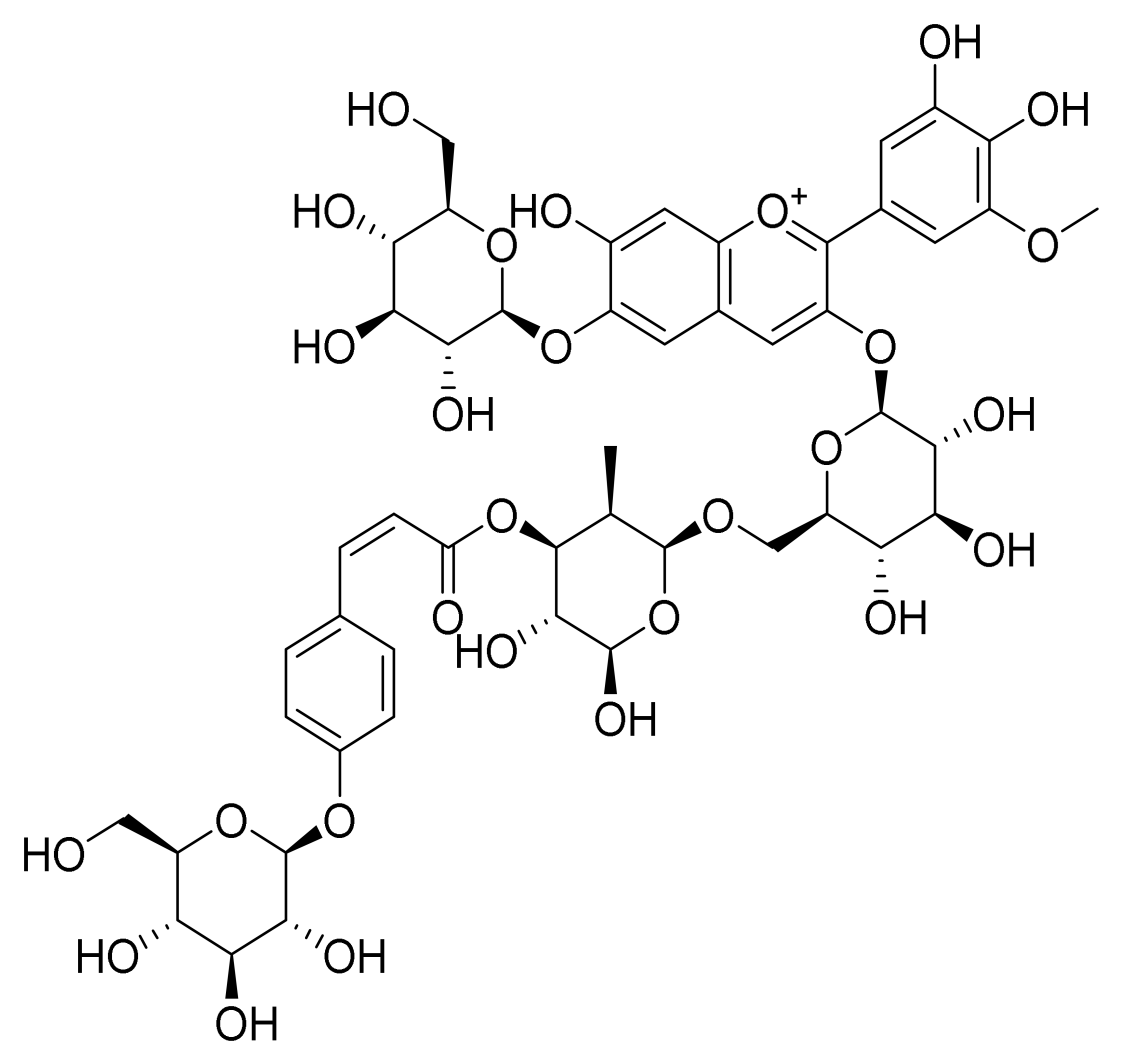			MS and NMR	[26]
3	petunidin 3-*O*-[6-*O*-(4-*O*-(4-*O*-(β-D-glucopyranosyl)-trans-*p*-coumaroyl)-α-L-rhamnopyranosyl)-β-D-glucopyranoside]-5-*O*-[β-D-glucopyranoside]	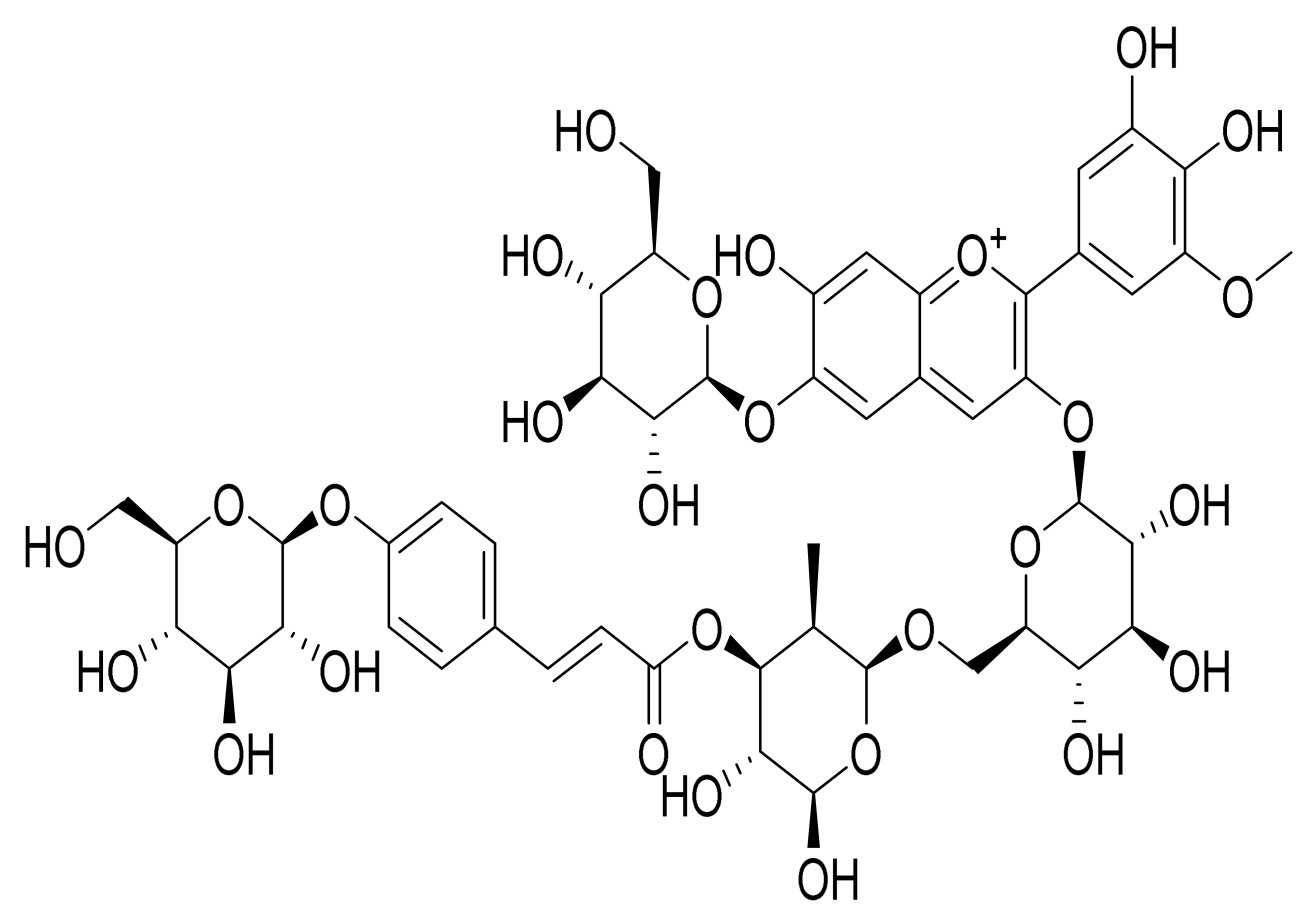			MS and NMR	[26]
4	petunidin 3-*O*-[6-*O*-(4-*O*-(cis-*p*-coumaroyl)-α-L-rhamnopyranosyl)-β-D-glucopyranoside]-5-*O*-[β-D-glucopyranoside]	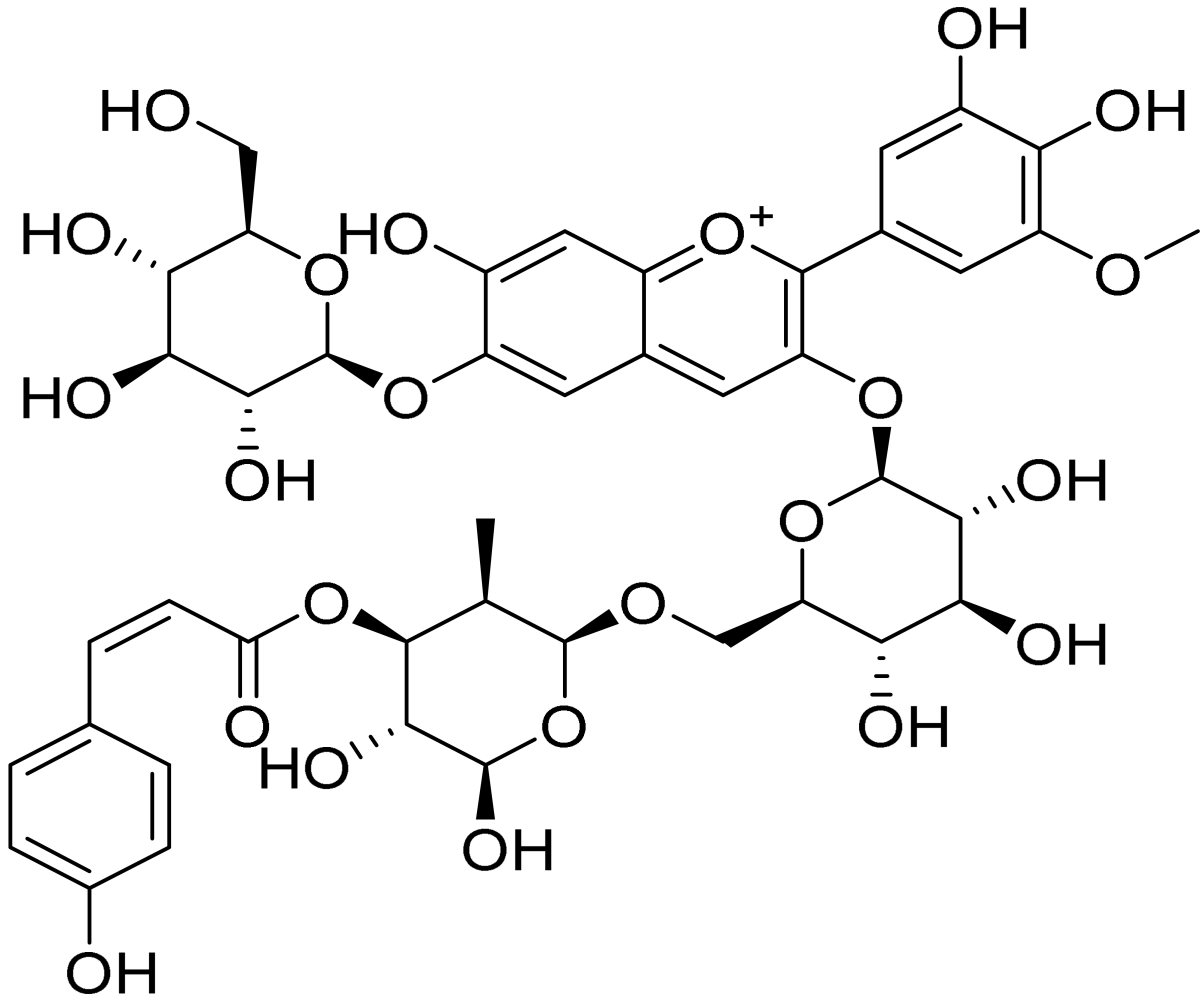			MS and NMR	[26]
5	petunidin 3-*O*-[6-*O*-(4-*O*-(trans-*p*-caffeoyl)-α-L-rhamnopyranosyl)-β-D-glucopyranoside]-5-*O*-[β-D-glucopyranoside]	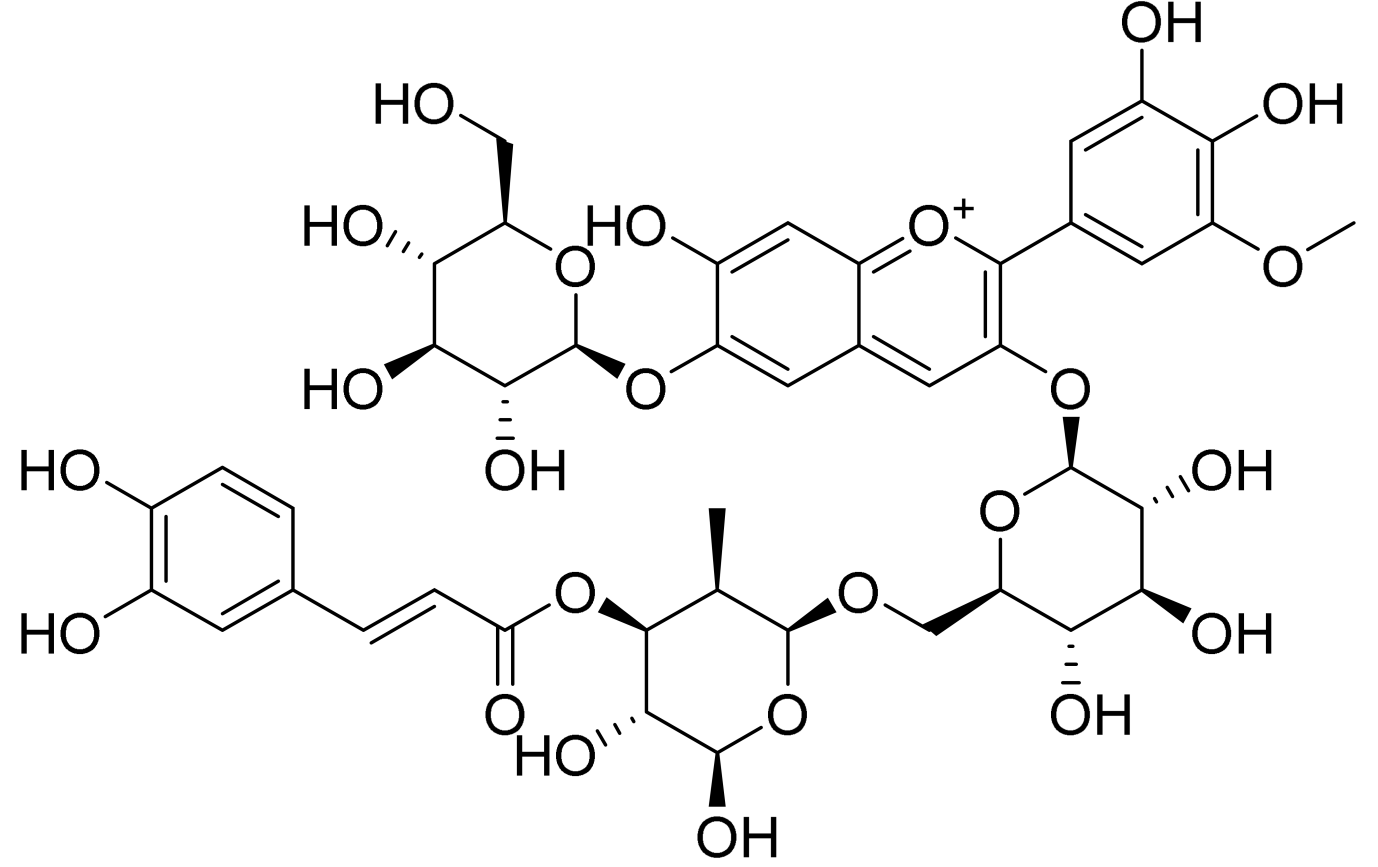			MS and NMR	[26]
6	petunidin 3-*O*-[6-*O*-(4-*O*-(trans-*p*-coumaroyl)-α-L-rhamnopyranosyl)-β-D-glucopyranoside]-5-*O*-[β-D-glucopyranoside]	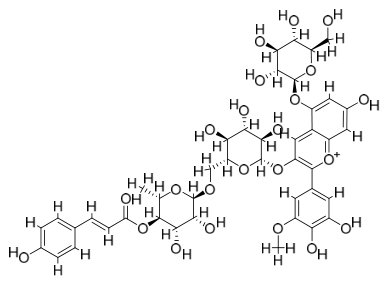			MS and NMR	[26]
7	petunidin-3-*O*-glucoside (maloyl)-5-*O*-glucoside	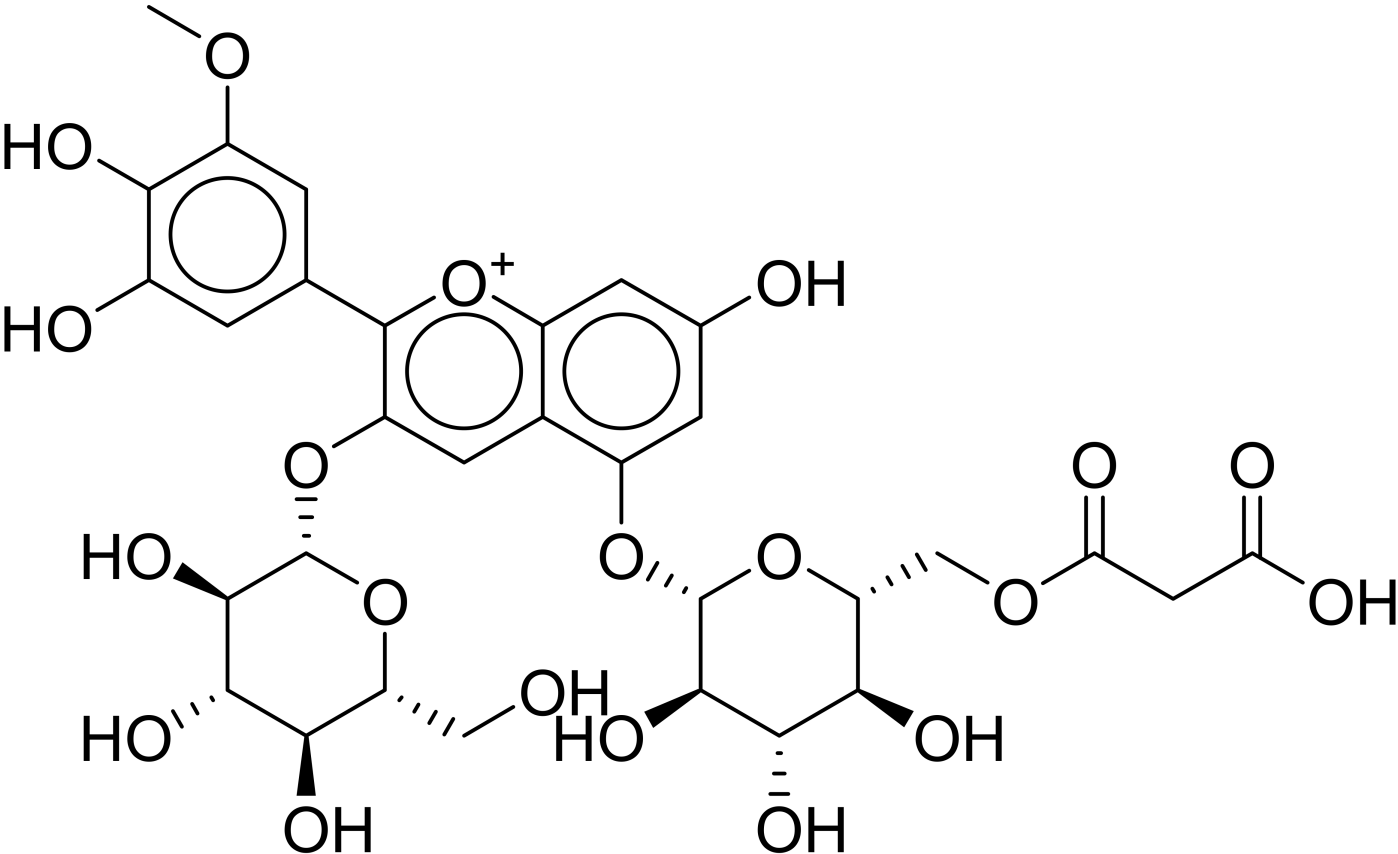			HPLC-DAD-ESI-MS and HPLC-ESI-ToF-MS	[27]
8	petunidin-3-*O*-galactoside-5-*O*-glucoside	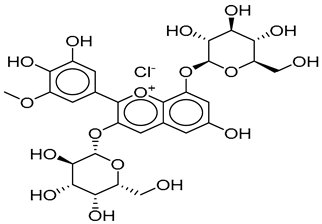			HPLC-DAD-ESI-MS and HPLC-ESI-ToF-MS	[27]
9	petunidin-3-*O*-glucoside (feruloyl)-5-*O*-glucoside	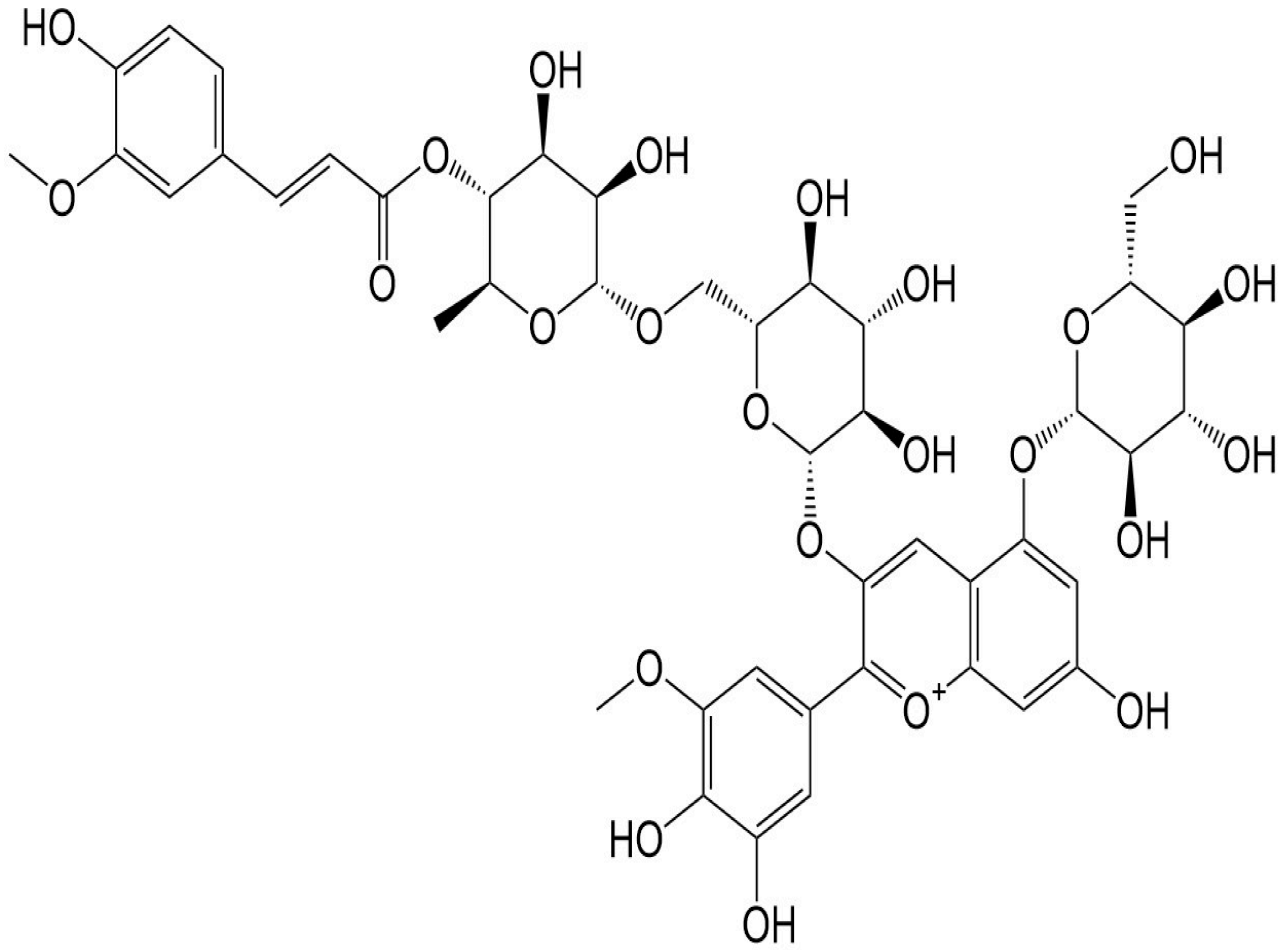			HPLC-DAD-ESI-MS and HPLC-ESI-ToF-MS	[27]
10	petunidin-3-*O*-glucoside-5-*O*-glucoside	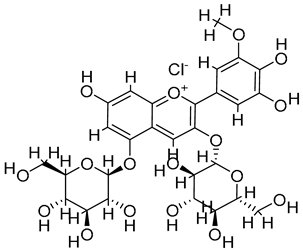			HPLC-DAD-ESI-MS and HPLC-ESI-ToF-MS	[27]
11	petunidin-3-*O*-rutinoside (caffeoyl)-5-*O*-glucoside	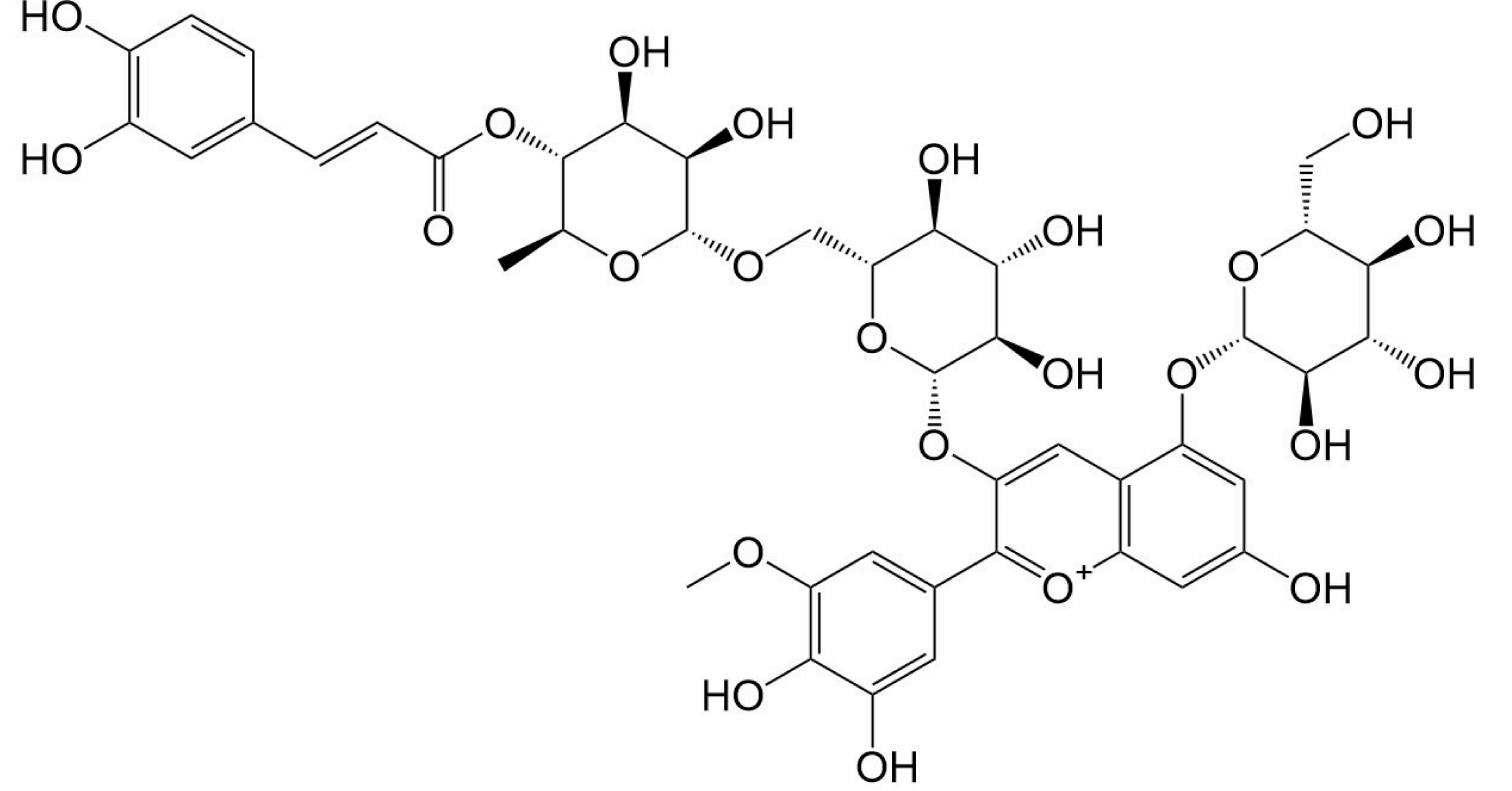			HPLC-DAD-ESI-MS and HPLC-ESI-ToF-MS	[27]
12	delphinidin 3-*O*-[6-*O*-(4-*O*-(trans-*p*-coumaroyl)-α-L-rhamnopyranosyl)-β-D-glucopyrano-side]-5-*O*-[β-D-glucopyranoside]	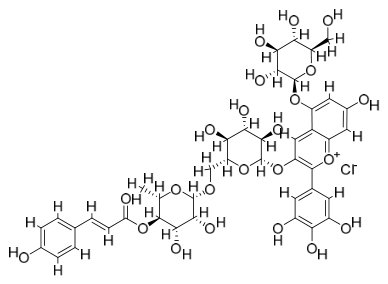	delphinidin	12.9%	MS and NMR	[26]
13	malvidin 3-*O*-[6-*O*-(4-*O*-*p*-coumaroyl-α-L-rhamnosyl)-β-D-glucopyranoside]-5-*O*-β-D-glucopyranoside	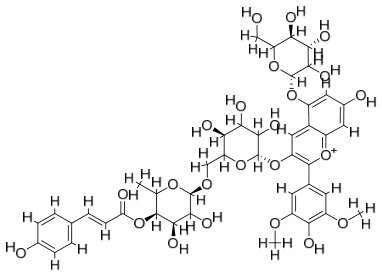	malvidin	4.4%	HPLC-DAD-ESI-MS	[28]
14	malvidin-3-*O*-rutinoside (cis-*p*-coumaroyl)-5-*O*-glucoside	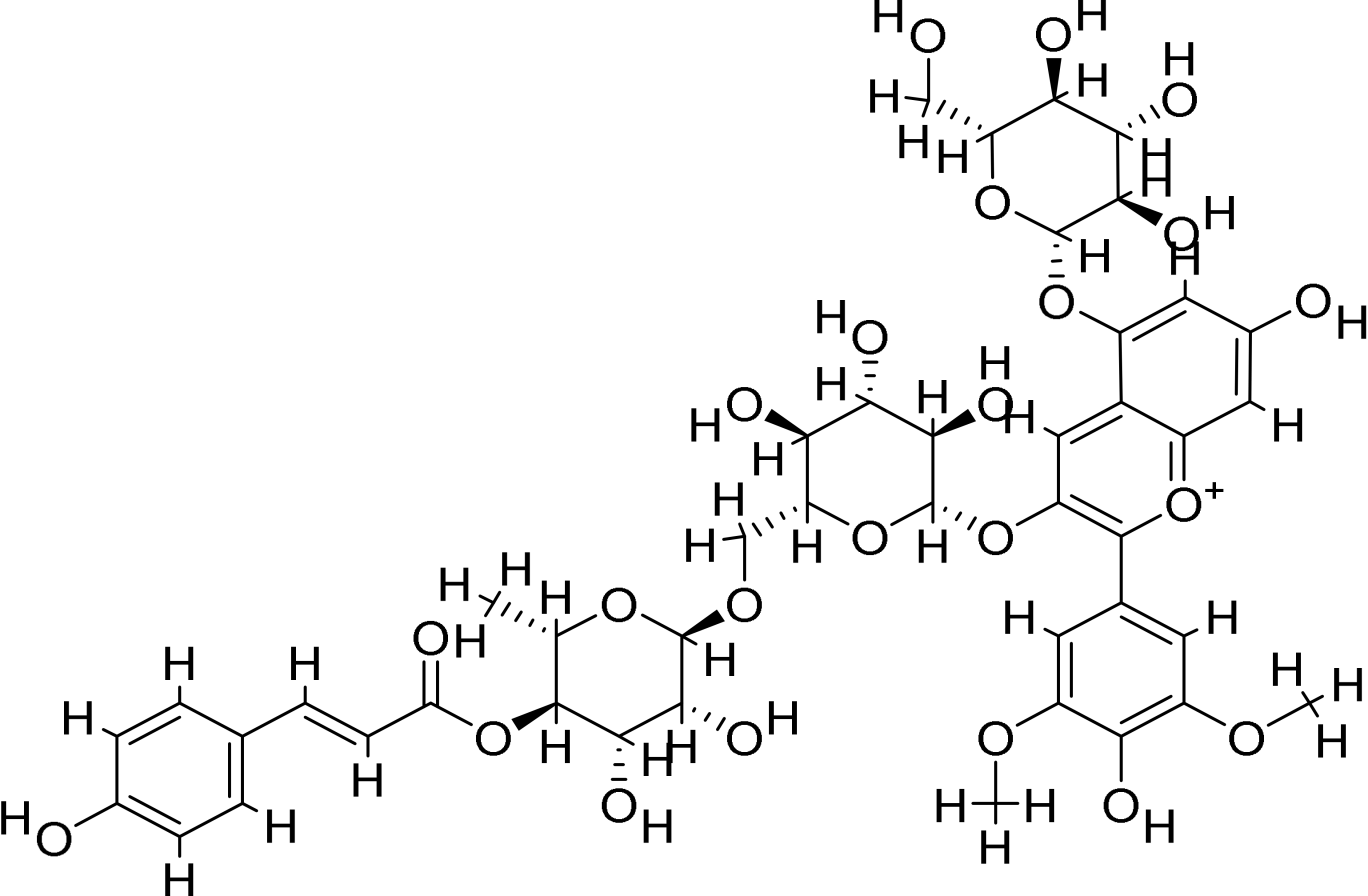			HPLC-DAD-ESI-MS and HPLC-ESI-ToF-MS	[27]

## Data Availability

No new data were created or analyzed in this study. Data sharing is not applicable to this article.

## Supplementary Materials

The following supporting information can be downloaded at: https://www.mdpi.com/article/10.3390/nu18193160/s1. Note S1: Full search strings for PubMed and Web of Science Core Collection.
