# Supplementary material for: Lycium ruthenicum as a Functional Food Resource: Chemical Composition, Nutritional Value, Health Benefits, and Biological Activities
Source: Nutrients. 2026 Sep 25;18(19):3160. doi: 10.3390/nu18193160 (PMC13666758; doi:10.3390/nu18193160)
Supplement: Supplementary file 1 [file nutrients-18-03160-s001.zip › nutrients-4540683-supplementary.pdf]

Supplementary Material

Lycium ruthenicum as a Functional Food Resource: Chemical Composition, Nutritional Value, Health Benefits, and Biological Activities

Note S1. Full search strings for PubMed and Web of Science Core Collection.

PubMed
("Lycium ruthenicum"[Title/Abstract] OR "black goji"[Title/Abstract] OR "black wolfberry"[Title/Abstract]) AND (anthocyanin*[Title/Abstract] OR polysaccharide*[Title/Abstract] OR composition[Title/Abstract] OR processing[Title/Abstract] OR bioactiv*[Title/Abstract] OR genomic*[Title/Abstract] OR "functional food"[Title/Abstract])

Web of Science Core Collection
TS=("Lycium ruthenicum" OR "black goji" OR "black wolfberry") AND TS=(anthocyanin* OR polysaccharide* OR composition OR processing OR bioactiv* OR genomic* OR "functional food")
